# IL-17A, a possible biomarker for the evaluation of treatment response in *Trypanosoma cruzi* infected children: A 12-months follow-up study in Bolivia

**DOI:** 10.1371/journal.pntd.0007715

**Published:** 2019-09-25

**Authors:** Clara Vásquez Velásquez, Graciela Russomando, Emilio E. Espínola, Zunilda Sanchez, Kota Mochizuki, Yelin Roca, Jimmy Revollo, Angelica Guzman, Benjamín Quiroga, Susana Rios Morgan, Roberto Vargas Ortiz, Alberto Zambrana Ortega, Eida Espinoza, Juan Eiki Nishizawa, Mohamed Gomaa Kamel, Mihoko Kikuchi, Shusaku Mizukami, Kesara Na-Bangchang, Nguyen Tien Huy, Kenji Hirayama

**Affiliations:** 1 Department of Immunogenetics, Institute of Tropical Medicine (NEKKEN), School of Tropical Medicine and Global Health, Nagasaki University, Sakamoto, Nagasaki, Japan; 2 Graduate School of Biomedical Sciences, Nagasaki University, Sakamoto, Nagasaki, Japan; 3 Departamento de Biología Molecular y Biotecnología, Instituto de Investigaciones en Ciencias de la Salud, Universidad Nacional de Asunción, Asunción, Paraguay; 4 Centro Nacional de Enfermedades Tropicales (CENETROP), Santa Cruz, Bolivia; 5 Programa Departamental de Control de Chagas del Ministerio de Salud, Santa Cruz, Bolivia; 6 Hospital Municipal Warnes "Nuestra Señora del Rosario", Santa Cruz, Bolivia; 7 Centro Médico Integral Siraní, Santa Cruz, Bolivia; 8 Faculty of Medicine, Minia University, Minia, Egypt; 9 Chulabhorn International College of Medicine, Thammasat University, Pathumthani, Thailand; 10 Department of Clinical Product Development, Institute of Tropical Medicine (NEKKEN), School of Tropical Medicine and Global Health, Nagasaki University, Nagasaki, Japan; Instituto de Ciências Biológicas, Universidade Federal de Minas Gerais, BRAZIL

## Abstract

**Background:**

The National Program for Chagas disease was implemented in Bolivia in 2006, and it greatly decreased the number of infections through vector control. Subsequently, a treatment regimen of benznidazole (BNZ) was started in seropositive school-age children living in certified vector control areas.

**Methods and findings:**

We conducted a 12-month follow-up study and seven blood samples were taken during and after the treatment. Serology, conventional diagnostic PCR (cPCR) and quantitative Real-time PCR (qPCR) were performed. Plasma Th1/Th2/Th17 cytokines levels were also determined. Approximately 73 of 103 seropositive children complied with BNZ, with three interruptions due to side effects.

To evaluate each individual’s treatment efficacy, the cPCR and qPCR values during the final 6 months of the follow-up period were observed. Among 57 children who completed follow-up, 6 individuals (11%) showed both cPCR(+) and qPCR(+) (non reactive), 24 (42%) cPCR(-) but qPCR(+) (ambiguous) and 27 (47%) cPCR(-) and qPCR(-) (reactive).

Within 14 Th1/Th2/Th17 cytokines, IL-17A showed significantly higher levels in seropositive children before the treatment compared to age-matched seronegative children and significantly decreased to the normal level one-year after. Moreover, throughout the follow-up study, IL-17A levels were positively co-related to parasite counts detected by qPCR. At the 12 months’ time point, IL-17A levels of non-reactive subjects were significantly higher than either those of reactive or ambiguous subjects suggesting that IL-17A might be useful to determine the reactivity to BNZ treatment.

**Conclusions:**

Plasma levels of IL-17A might be a bio-marker for detecting persistent infection of *T*. *cruzi* and its chronic inflammation.

## Author summary

Chagas is a zoonosis endemic in 21 Latin American countries caused by *T*. *cruzi*. Results of common Benznidazole (BNZ) treatment vary by infection phase, treatment period, and dosage. In Bolivia, the national Chagas program controls vector distribution in different regions of the country. The program began BNZ treatment in school-age children from infestation-free endemic areas. Lack of information regarding follow-up and efficacy in children with recent chronic Chagas makes treatment failure difficult to detect in endemic areas. The present study aimed to estimate parasite DNA in blood through quantitative real-time and conventional PCR (qPCR, cPCR), and observe Th1/Th2/Th17 cytokine profiling during a 12-month follow-up in Bolivia school children. Results showed persistence of low, substantial amounts of *T*. *cruzi* DNA, and significantly higher IL-17A levels in the seropositive group before treatment than the seronegative group, which decreased to seronegative levels one year later. Of 57 treated, 6 showed cPCR positive results 6 months after treatment and were diagnosed as definitely non-reactive (10.5%). The six non-reactive individuals showed significantly higher levels of IL-17A at 12 months than residual reactive (cPCR negative, qPCR negative) and ambiguously reactive (cPCR negative, qPCR positive) groups, indicating that IL-17A might be a biomarker for non-reactive to BNZ.

## Introduction

An estimated seven million people are infected with *Trypanosoma cruzi*, a parasite that causes Chagas disease worldwide, mostly in Latin America. The transmission occurs when humans come in contact with infected feces of blood-sucking *triatomine* bugs, or through blood transfusions using blood from infected donors, vertical transmission, organ transplantation, contaminated food or laboratory accidents [1]. Without any appropriate treatment, almost all the infected patients develop a chronic phase in which the parasites systematically infect the heart and digestive organs [2, 3]. Two drugs are currently available for Chagas treatment, nifurtimox, and benznidazole (BNZ), which are effective as anti-parasitic for *T*. *cruzi*, however, their prolonged regimens and undesirable side effects are the main drawbacks in their employment [4–9].

To consider a patient cured of chronic *T*. *cruzi*, the “negativization” of two different serological assays should be achieved [10–12]. However, long follow-up is necessary before this “negativization” of serological assays can be observed, making current serological assays difficult to use as monitoring tools during the first year of post-treatment especially in chronic cases. In a placebo-controlled trial in a rural area of Brazil on school children with endemic Chagas disease, 55.8% of treatment efficacy was observed by a negative seroconversion after a three-year follow-up [13]. Other studies have reported around 60–80% response in children under 15 years old with chronic infection treated with BNZ and assessed by the conventional serology in a long-term follow-up [14–17].

Therefore, the application of polymerase chain reaction (PCR) to detect *T*. *cruzi* directly in blood samples has become an alternative method for the early evaluation of infection and treatment efficacy in different settings in Latin America (Brazil, Argentina, Colombia, Bolivia, and Chile) and Spain [18–23]. Nevertheless, the fluctuations of circulating *T*. *cruzi* in chronic Chagas disease patients might affect PCR sensitivity and sampling at different time points of the follow-up may be needed. In this context, a molecular strategy to quantify *T*. *cruzi* DNA in peripheral blood samples as quantitative real-time PCR (qPCR), is a key tool for monitoring parasitological response during follow-up of the treatment in chronic Chagas disease patients.

Additionally, it has been suggested that BNZ is involved in the host immune regulation in Chagas by reducing the development of severe disease [24], leading to T- and B-cell activation with the participation of type 1-modulated cytokine pattern and by increased levels of IFN-γ production that improves the chemotherapy efficacy [24–26]. However, no clear relation to the effectiveness of the treatment with BNZ and cytokine profile was determined.

Currently in Bolivia, the National Chagas Program has been successful in certifying vector controlled areas where the *Triatoma infestans* residual infestation is less than 3% in parts of the country [10]. Therefore, the treatment program has initiated in these certified areas a BNZ regimen of 5 mg/kg/day for 60 days in seropositive school-aged children. Accordingly, if children are ELISA positive, they receive BNZ for free [10]. However, lack of information regarding treatment follow-up and efficacy in children makes it difficult to detect treatment failure.

The present study aimed to estimate the blood levels of parasitemia using quantitative real-time PCR (qPCR) during a 12-month follow-up in school children with recent chronic Chagas from the Bolivia National Chagas Program. The levels of Th1/Th2/Th17 cytokines were also estimated during the follow-up and compared to *T*. *cruzi* molecular detection outcomes to find out patterns of response to BNZ treatment.

## Methods

### Recruitment and participants

From April 2012 to March 2015, a total of 126 children aged 4–15 years old originally from Santa Cruz, Bolivia were identified through rapid immunochromatographic test (RIT) Chagas STAT-PAK (RIT, Chembio Diagnostic Systems, Medford, NY, USA) for a qualitative detection of antibodies to *T*. *cruzi* by the Departmental Program of Chagas Control in six regional health facilities: Warnes Municipal Hospital “Nuestra Señora del Rosario” (Warnes, Santa Cruz, Bolivia), Health care center Satelite Norte (Satelite Norte) in Warnes, Health care center “Villa de Cochabamba” in Montero (Montero), Portachuelo Hospital “San Jose Obrero” (Portachuelo), Health care center “18 de Marzo, Santa Cruz” (Santa Cruz), and Centro Nacional de Enfermedades Tropicales (CENETROP, Santa Cruz).

Samples from children with positive RIT were sent from the aforementioned outpatient clinics to CENETROP, the national reference center for the diagnosis of Chagas, for the confirmation of *T*. *cruzi* infection by Wiener Recombinant 3.0 Chagatest Enzyme-linked immunosorbent assay (ELISA, Wiener Laboratorios, Santa Fé, Argentina), Chagas Polychaco Indirect Haemagglutination test kit (IHA, Lemos S.R.L. Laboratories, Santiago del Estero, Argentina), and Indirect Immunofluorescence assay (IIF, Immunofluor Chagas, Biocientífica, Argentina) [27]. Calculations of the cutoff values and evaluation of the test results were performed as described in the respective sections of each manual.

The inclusion criteria were as follows: permanent residents in the towns where the study was performed, no underlying chronic disease, no history of prior treatment for the Chagas disease, having the consent of their parents and living in a location free of an infestation of *Triatoma infestans*. Those having a history of hypersensitivity to medications or a history of prior treatment failure were excluded. If all three tests were positive and complied with the inclusion criteria, they were asked to participate in the study and informed consent was obtained.

**Fig 1:**
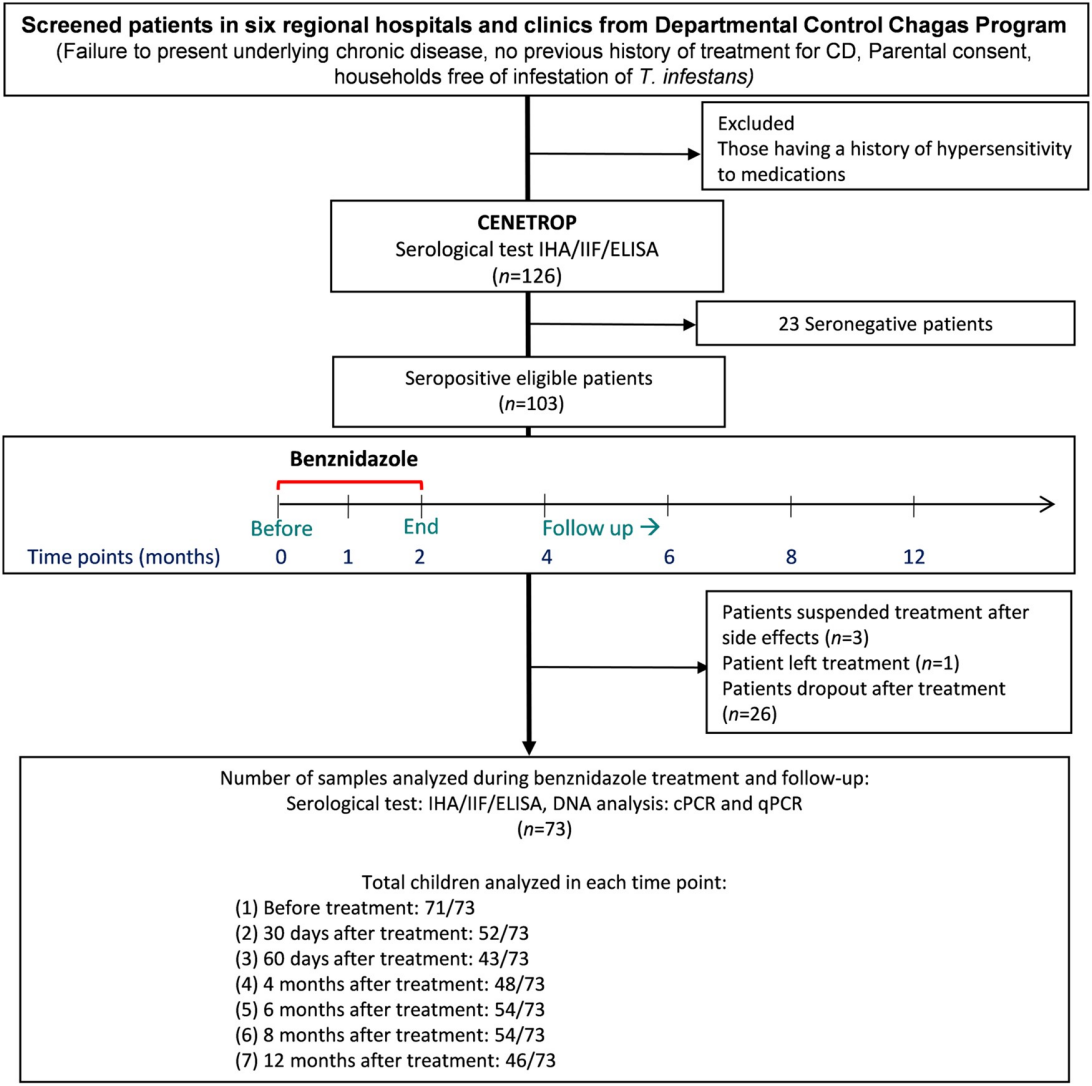
The flow diagram of study design, patient selection, treatment and follow-up. CD, Chagas Disease. *T*., *Triatoma*. IHA, Haemagglutination test. IIF, Indirect Immunofluorescence test. ELISA, Enzyme-Linked ImmunoSorbent Assay. cPCR, conventional PCR. qPCR, quantitative real-time PCR.

Confirmed seropositive patients were treated with 5 mg/kg BNZ every 12 hours for a period of 60 days according to the National Program [10]. Before starting BNZ, clinical examination, laboratory blood tests, and electrocardiogram were performed. All patients were followed-up after the initiation of therapy at days 30 and 60 with a recorded paper of the date, dose, time of the administration of BNZ, and the presence of any unexpected symptoms during the 60 days regimen. Additionally, blood samples were collected at months 4, 6, 8 and 12 after the initiation of benznidazole (Fig 1).

### Samples and DNA extraction

Blood samples with EDTA (5–7 ml) were collected seven times for each patient including (1) before treatment, (2) 30 days, (3) 60 days, (4) 4 months, (5) 6 months, (6) 8 months, and (7) 12 months after the treatment initiation. After extraction, plasma was separated by centrifugation at 3,000 × g for 15 minutes and stored at -80°C until analysis.

Aliquots for cell fraction were frozen in CENETROP at -20°C and then shipped to the laboratory at the Instituto de Investigaciones en Ciencias de la Salud, Universidad Nacional de Asunción in Paraguay (IICS) on dry ice for DNA extraction using QIAamp DNA Blood Maxi kit (QIAGEN, GmbH, Hilden, Germany) in accordance with the instruction manual. A total volume of 1 ml of extracted DNA was obtained from each sample and 500 microliters were shipped on dry ice to the Department of Immunogenetics, Institute of Tropical Medicine (NEKKEN), Nagasaki University, Japan, followed by their maintenance at -30°C.

### Measurement of BNZ levels in plasma samples

Plasma samples available from each seropositive patient before treatment, 30 and 60 days after the initiation of treatment (3-time points) were analyzed. Concentrations of BNZ in all plasma samples were measured at the Center of Excellence in Pharmacology and Molecular Biology of Malaria and Cholangiocarcinoma, Thammasat University (Thailand), using high-performance liquid chromatography (HPLC) [28]. Briefly, 10 μl of the internal control (300 μg/ml of diazepam) was spiked into 400 μl of plasma. The sample extraction was performed twice using 1 ml of diethyl ether. Following centrifugation (2,500 × g, 15 minutes), the upper organic phase was transferred to a clean tube and dried at 40°C. The residue was reconstituted with 150 μl HPLC mobile phase (water/acetonitrile at the ratio of 60∶40 v/v) and 30 μl was injected onto the column. The HPLC chromatographic system consisted of Hypersil GOLD C18 RP column (Thermo Fisher Scientific Inc.) and the mobile phase running at a flow rate of 1.0 ml/min, with UV detection at 324 nm. The limit of quantitation (LOQ) for benznidazole was 0.1 μg/ml. The method was linear in a range of 0.1–20.0 μg/ml.

### Conventional PCR (cPCR)

The Forward and Reverse primers set, 121 (5’-AAATAATGTACGGGKGAGATGCATGA-3’) and 122 (5’-GGTTCGATTGGGGTTGGTGTAATATA-3’) were used for amplification of minicircle kinetoplast DNA[29], to obtain 330 bp amplicon. Each sample was tested in a final volume of 25 μl including 1 μl (10–15 ng) of extracted DNA, 0.5μl (10 pmol/μl) of the primers (121,122), and 2.5 μl of 10× buffer containing 25 mM MgCl_2_, 2.5 mM of dNTPs, and 2 unit of Takara Ex Taq enzyme (Takara Bio. Inc., Shiga, Japan). The PCR amplification was performed using Thermal cycler (Biometra, GmbH, Germany) programmed at 94 °C for 3 minutes as initial denaturation, followed by 2 cycles at 97.5°C for 1 minute and 64°C for 2 minutes, 33 cycles at 94°C for 1 minutes and 62°C for 1 minute, and a final extension for 10 minutes at 72 °C. The product from PCR was confirmed by 2% agarose gel electrophoresis [29].

### Quantitative real-time PCR (qPCR)

Detection of *T*. *cruzi* was performed by amplifying the DNA satellite sequence (GenBank: K01773.1) with primers TCZNTAQF (5'-GCTCTTGCCCACACGGGTG-3'), TCZNTAQR (5'-AAGCAGCGGATAGTTCAGGGT-3'), and TaqMan MGB probe TCZ-FAM (5'-ACTCGGCTGATCGTT-3'). As an internal amplification control, glyceraldehyde 3-phosphate dehydrogenase intron C (GAPDH-C) was detected by primers GAPDH-C-36F (5'-GCCCCTTCATACCCTCACGTA-3'), GAPDH-C-141R (5'-TGACAAGCTTCCCGTTCTCAG-3') and TaqMan probe GAPDH-VIC (5'-ATGTTCCAATATGATTCCAC-3') designed by the primer express Software v3.0 (Thermo Scientific, Waltham, MA, USA) (S1 Table). To make a qPCR standard curve for estimating parasite DNA amount and its equivalent parasite number in each DNA test sample extracted from whole blood, DNA was extracted from peripheral blood of a seronegative healthy individual (Japanese with no history of travel to an endemic area), and from culture form of *T*. *cruzi* epimastigotes of the Bolivia DR31 strain (isolated at Santa Cruz, Bolivia, previously characterized as TcV (former TcIId) [30, 31]. *T*. *cruzi* DNA was obtained from 1 × 10^5^ epimastigote cells/ml grown in a liver infusion tryptose medium (LIT) [32] using QIAamp DNA purification (QIAGEN, GmbH, Germany). *T*. *cruzi* stocks of Bolivian DR31 strain were provided by Dr. Tetsuo Yanagi at National Bioresource Project, NEKKEN, Nagasaki University, Japan. Seronegative DNA and parasite DNA were stored at -30°C until use.

For the amplification of qPCR, a 96 well-plate was used, which contained the standard curve for *T*. *cruzi* and GAPDH-C target in duplicates, 2 non-template controls with *T*. *cruzi* and GAPDH-C primers as a negative control to detect the contamination at any stage of the procedure in each run. The qPCR reactions were carried out with 2 μl of DNA template, 10 μl Hot Start Premix Ex Taq 2X Probe master mix (Takara Bio. Inc., Shiga, Japan), using 5 pmol of *T*.*cruzi* forward and reverse primer (TCZNTAQF, TCZNTAQR) and 8 pmol of TCZ-FAM, 4 pmol of human forward and reverse primer (GAPDH-IntronC-36F, GAPDH-IntronC-141R) and 8 pmol of GAPDH-VIC, 0.4 μl of 50X Rox Reference DyeII in a final volume of 20 μl. The optimal cycling conditions were a first step of 30 sec at 95°C followed by 50 cycles of 5 sec at 95°C and 32 sec at 62°C.

Each sample was considered valid when the internal control GAPDH-C was efficiently amplified in each run, and positive for *T*. *cruzi* satellite DNA when detectable for *T*. *cruzi* target in a cycle threshold (Ct) below 45 cycles (Ct 30 = 300 fg of *T*. *cruzi* DNA). The detection limit cut-off to determine negative for *T*. *cruzi* was established once the Ct value below 45 was obtained using the DNA extracted from the blood of two healthy Japanese with no background of traveling to South America after replicating the experiment 30 times from each sample. Because the Ct values sometimes showed close to the upper limit, all the patients’ samples were carried out in replicate experiments to a total of five times.

qPCR was performed sequentially in IICS and then NEKKEN to ensure reproducibility. The amplifications were carried out in an Applied Biosystems 7500 Real-Time PCR System (Thermo Scientific, Waltham, MA, USA) device in NEKKEN and CFX96 Touch Real-Time PCR Detection System (BIO-RAD Lab, Hercules, CA, USA) device in IICS. Ct values and quantity data were expressed as arithmetic means ± standard deviation using 7500 Software version 2.0.6 (Thermo Scientific, Waltham, MA, USA).

### Efficacy end-points

Reactive to BNZ (cPCR(-) qPCR(-)) was defined as *T*. *cruzi* DNA absence on cPCR and qPCR in more than 2 sampling points during a 6–12 months period. Non-reactive (cPCR(+) qPCR(+)) was defined as the presence of *T*. *cruzi* DNA on cPCR and/or qPCR(+) at any sampling point during a 6–12 months period. Ambiguous (cPCR(-) qPCR(+)) was defined as positive qPCR in 2 sampling points during a 6–12 months period with a negative cPCR at any sampling point after 6 months. Children who failed to complete follow-up (unless qPCR after treatment was positive), dropped out due to side effects, and who had negative qPCR in only one-time point during a 6–12 months period were excluded.

### Cytokine quantification

Plasma concentration levels of the following cytokines: tumor necrosis factor (TNF)-α, interferon (INF)-γ, interleukin (IL)-2, IL-4, IL-5, IL-10, IL-13, IL-9, IL-17A, IL-17F, IL-6, IL-21, IL-22, and IL-23 were measured. All above cytokines except for IL-23 were measured using the LEGENDplex Human Th cytokine Capture Beads multi-analyte flow assay kit (Cat.740118). IL-23 was measured using LEGENDplex Human IL-23 assay kit (Cat.740108). For additional confirmation of the IL-17A, the LEGENDplex Human IL-17A assay kit (Cat.740048) was performed as per manufacturer’s instructions (BioLegend, San Diego, CA, USA). Data acquisition was obtained using FACSVerse (BD Biosciences) flow cytometer and fluorescence was performed using the FACSsuite (BD Biosciences) software. The LEGENDplex 7.0 and 8.0 Data Analysis software were used for data analysis.

### Statistical analysis

A non-parametric statistical analysis of the data was applied due to the non-normal distribution of the data using the skewness and kurtosis tests. Comparisons were performed using the Kruskal-Wallis test and Dunn’s post-test for selected multiple comparisons. Mann-Whitney or Wilcoxon signed-rank test was applied to determine significant differences between medians of parasitic loads by qPCR and IL concentration levels prior to treatment and follow-up. The correlation coefficient between the parasitemia and cytokines concentration was calculated by the Spearman rank test.

Analyses were carried out using GraphPad Prism software 8 for Windows 64-bit, version 8.0.2 (GraphPad Software, La Jolla California, USA). A *P*-value of less than 0.05 was considered statistically significant. Furthermore, LOESS smoothing was applied using ggplot2 in RStudio (version 1.0.153) to plot parasitemia by qPCR in relation to time follow-up points.

### Ethics statement

The experimental protocol was approved by the Institutional Ethical Review Committee of NEKKEN, Nagasaki University, Japan (No. 12091097) and CENETROP, Bolivia. The written informed consent was obtained from parents or guardians on behalf of children. All the methods were conducted in accordance with the relevant approved regulations, guidelines, and declarations of Helsinki.

## Results

### Patients’ characteristics

In total, 126 children were positive using RIT in the six regional health facilities: Warnes (male = 12, female = 20, mean age 8.9 years), Satellite Norte (male = 17, female = 12, mean age 9.1 years), Montero (male = 12, female = 17, mean age 9.1 years), Portachuelo (male = 22, female = 8, mean age 8.9 years), Santa Cruz (male = 2, female = 3, mean age 12.2 years), and CENETROP (female = 1, 5 years old). All were referred to CENETROP for confirmation using serological tests as described in the methods section. As a result, 103 children without any acute symptoms, clinical laboratory abnormalities nor typical ECG alteration were confirmed positive by the three serology assays, and 23 were seronegative (Fig 1).

Followed by the National Chagas Program, 103 seropositive children started the treatment with benznidazole. Three patients (3%) interrupted therapy due to side effects of the medication and one dropped out. There were 26 children lost to follow-up after the treatment, thus 73 cases successfully came back to the clinic for analysis at least once during 2 to 12 months period after initiation of the treatment. The average attendance per patient during each periodic follow-up after the initiation of treatment was 69%. The attendance rates were 71% (52/73), 59% (43/73), 66% (48/73), 74% (54/73), 74% (54/73), and 63% (46/73) for 30 days, 60 days, and 4, 6, 8, and 12 months respectively (Fig 1).

### Side effects and plasma level of BNZ

We observed 14 children with symptoms related to side effects during the treatment but the majority of cases were mild. The most frequent adverse events were dermatological events in 6 out of 72 individuals (8.3%, mainly rash), followed by gastrointestinal disorders (5.5%, 4/72), headache (2.8%, 2/72) and joint pain (1.4%, 1/72). After the adverse events disappeared, all the individuals were able to complete their treatment regimen. One case with Stevens-Johnson syndrome was observed in a 10-year old girl at day 21 of BNZ starting with rash and progressing to persistent abdominal pain, vomiting, and a generalized red rash. An abdominal ultrasound revealed hepatosplenomegaly and gall bladder edema, thus she was transferred to the local hospital for the intensive care and the treatment was discontinued. To verify the absorption of BNZ during administration, BNZ levels in plasma 1 to 2 hours after taking a morning dose were recorded on day 30 (n = 56) and day 60 (n = 41) and the means (±SDs) were 5.4 (±3.7) and 3.1 (±4) μg/ml, respectively (S2a Fig).

### One-year follow-up of *T*. *cruzi* DNA fragments

**Fig 2:**
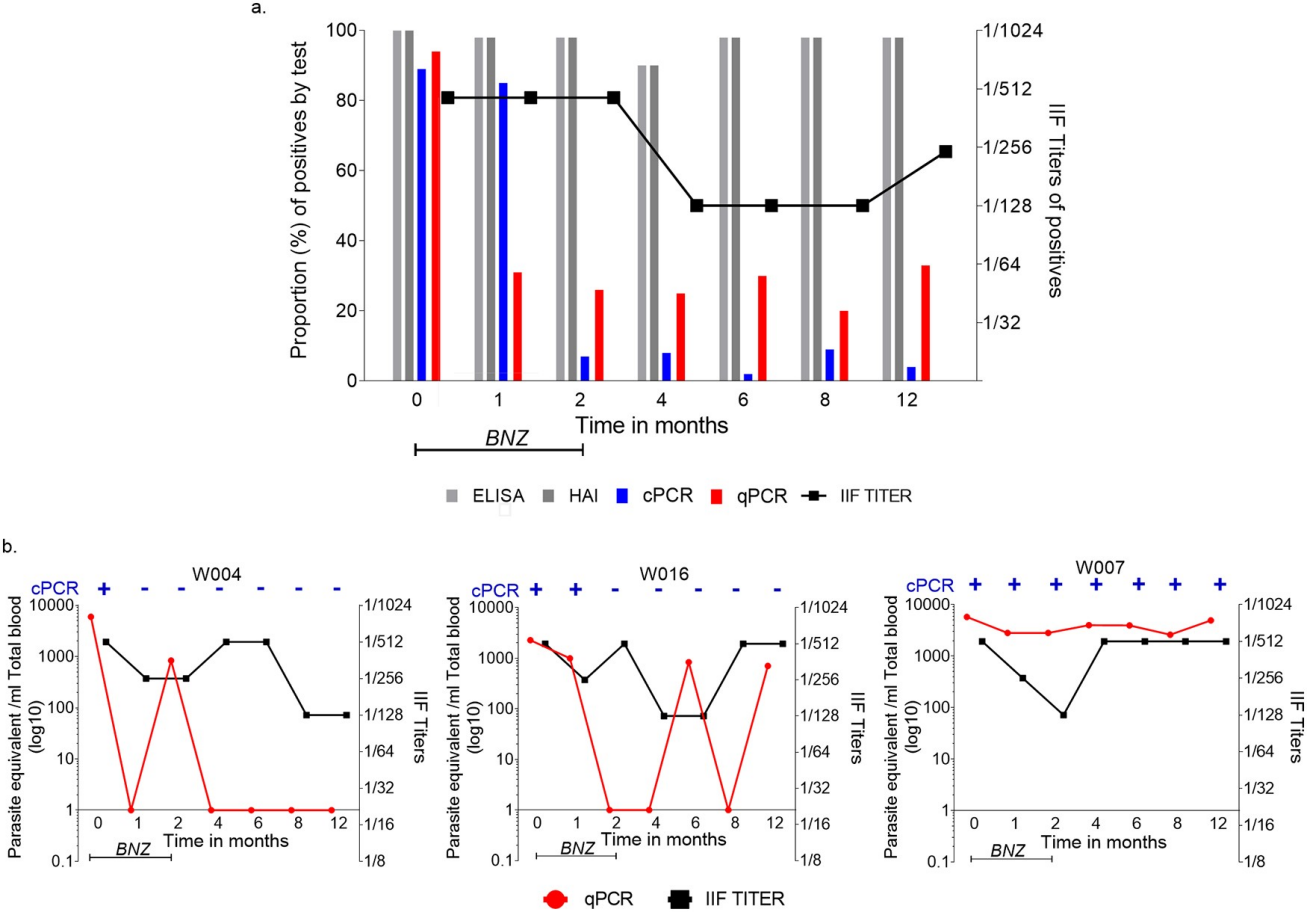
Follow-up in *Trypanosoma cruzi* seropositive children in the course of the 12-month after treatment with Benznidazole. a. The proportion of positives by ELISA, IHA, IIF, cPCR and qPCR. ELISA and IHA positive means reactive when tested by Wiener Recombinant 3.0 Chagatest ELISA and Chagas Polychaco IHA, respectively. IIF titers were considered reactive when fluorescence was observed at a 1:20 or higher dilution by Immunofluor Chagas test. cPCR Positive when agarose gel show 330 bp *T*. *cruzi* kDNA specific fragment amplification. qPCR Positive when *T*. *cruzi* parasite load were determined by the qPCR method as described at methods (negative by qPCR = Ct value above 45). b. Representative profile of IIF titers, cPCR, and qPCR in three *T*. *cruzi* seropositive children. W004: An individual with a decrease in the positive results by detection of DNA copies of *T*. *cruzi* by cPCR and qPCR were observed, even though anti-*T*. *cruzi* antibodies persisted; W016. Individual with decreased after 30 days of BNZ by detection of DNA copies of *T*. *cruzi* by cPCR, qPCR parasitemia, and anti-*T*. *cruzi* antibodies IIF titers persisted positive; W007. Individual with positive results by detection of DNA copies of *T*. *cruzi* by cPCR, qPCR and anti-*T*. *cruzi* antibodies after BNZ. cPCR is indicated by the color blue, positive (+) and negative (-); red circles represent median results from quintuples measures of DNA copies of parasite equivalent in 1 ml of total blood (log10) by qPCR; black square represents IIF titers. ELISA, Enzyme-Linked ImmunoSorbent Assay. IHA, Haemagglutination test. IIF, Indirect Immunofluorescence test. cPCR, conventional PCR; qPCR, quantitative real-time PCR; IIF titers, indirect immunofluorescence Titers. BNZ, treatment with Benznidazole.

In the course of the 12-month follow-up, a decrease in the positive results by detection of DNA copies of *T*. *cruzi* by cPCR and qPCR were observed, even though anti-*T*. *cruzi* antibodies (ELISA, IIF, and IHA) persisted (Fig 2a). The proportion of positives with ELISA, IHA and IIF was around 97% after starting BNZ (Fig 2a, S2 Table) and only one patient seroconverted after BNZ (S3 Table). The proportion of patients with positive cPCR presented a decrease from 89% (63/71) before treatment to 4% (2/46) after 12 months follow-up (Fig 2a, S2 Table). Through qPCR, the proportion of positives 94% (67/71) before the treatment decreased to 31% (16/51) after 30 days of treatment and stayed a similar proportion during the 12-month follow-up (Fig 2a, S2 Table).

**Fig 3:**
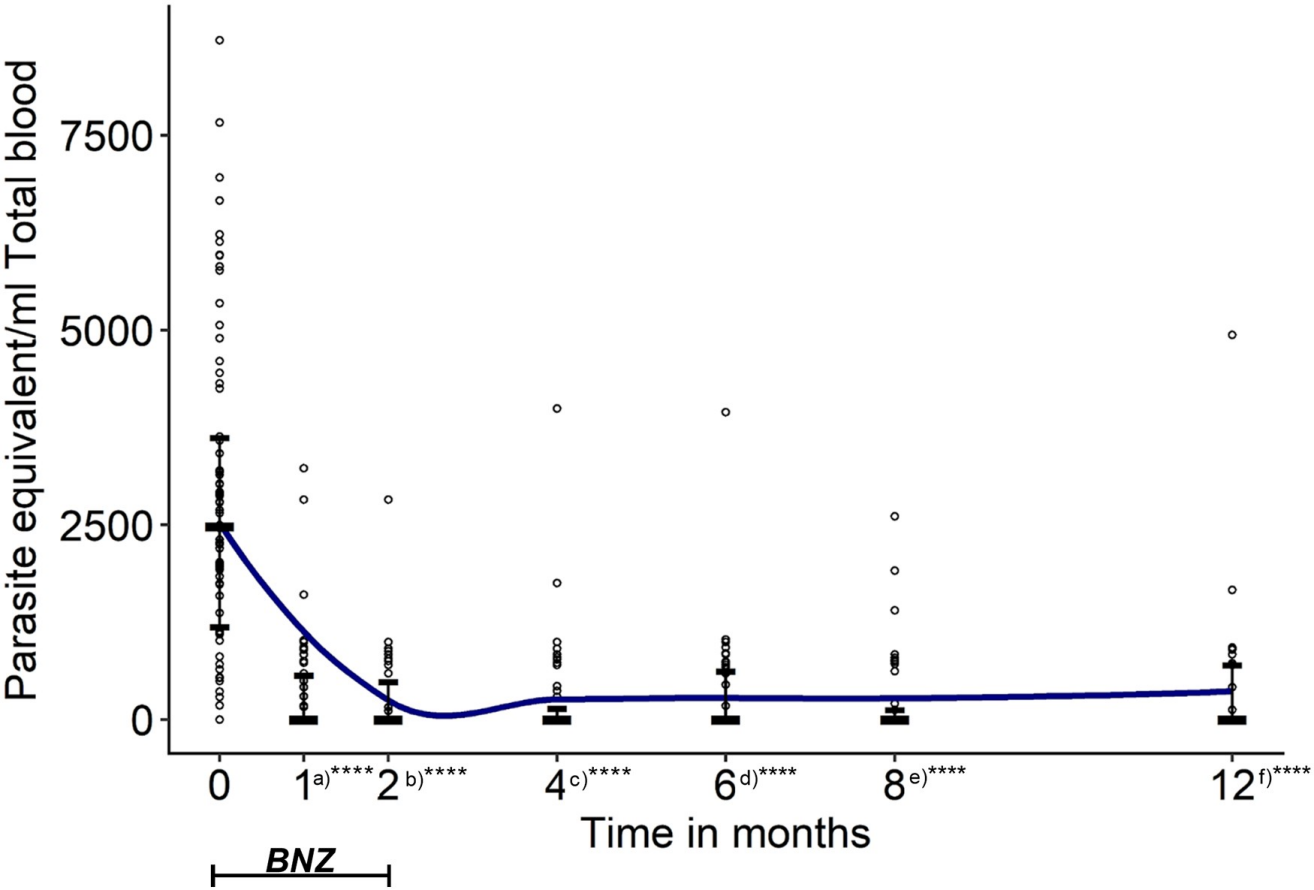
Before and post-treatment median of parasitemia of *T*. *cruzi* among recent chronic Chagas children measured by qPCR. The estimated parasite equivalent loads in 1 ml of total blood from children in each follow-up time points for 12 months. Association was determined between two groups by the nonparametric Mann-Whitney test (****P<0.0001): a) before treatment versus after 30 days of treatment; b) before treatment versus after 60 days of treatment; c) before treatment versus after four months of treatment; d) before treatment versus after 6 months of treatment; e) before treatment versus after eight months of treatment; f) before treatment versus after 12 months of treatment. Lines represent median and IQ. Each dot represents DNA copies of parasite equivalent in one ml of total blood by qPCR. Loess curve is shown in color blue using a bandwidth of one. The graph was constructed using the ggplot2 package in the R program. BNZ, treatment with Benznidazole.

We monitored the parasite loads of *T*. *cruzi* in the blood samples available in periodic follow-up during the 12 months using qPCR as summarized in S3 Table. qPCR was able to detect parasitemia during the follow-up even when cPCR became negative (Fig 2b (W016 and W007), S3 Table). The qPCR parasitemia levels during the follow-up were significantly lower when compared with the starting point of the treatment (Kruskal–Wallis test; *P*<0.0001) (Fig 3).

The available subjects at 6 months showed a significant decrease in parasitemia when compared with parasitemia level prior to treatment (*P*<0.0001). However, 30% (16/54) of children had low but significant amount of *T*. *cruzi* DNA copies (median: 788.5 (2.896 log_10_) parasites/ml; Ct median 38.35). Subjects available at 8 and 12 months showed a similarly significant decrease of parasitemia when compared with level prior to treatment (*P*<0.0001), with 20% (11/54) of the children with a significant amount of *T*. *cruzi* DNA copies (median: 794 (2.90 log_10_) parasites/ml whole blood; Ct median 38.3) at 8 months, and 33% (15/46) (median: 836 (2.92 log_10_) parasites/ml whole blood; Ct median 38) at the end of the follow-up (12 months) (Fig 3, S2 Table).

**Fig 4:**
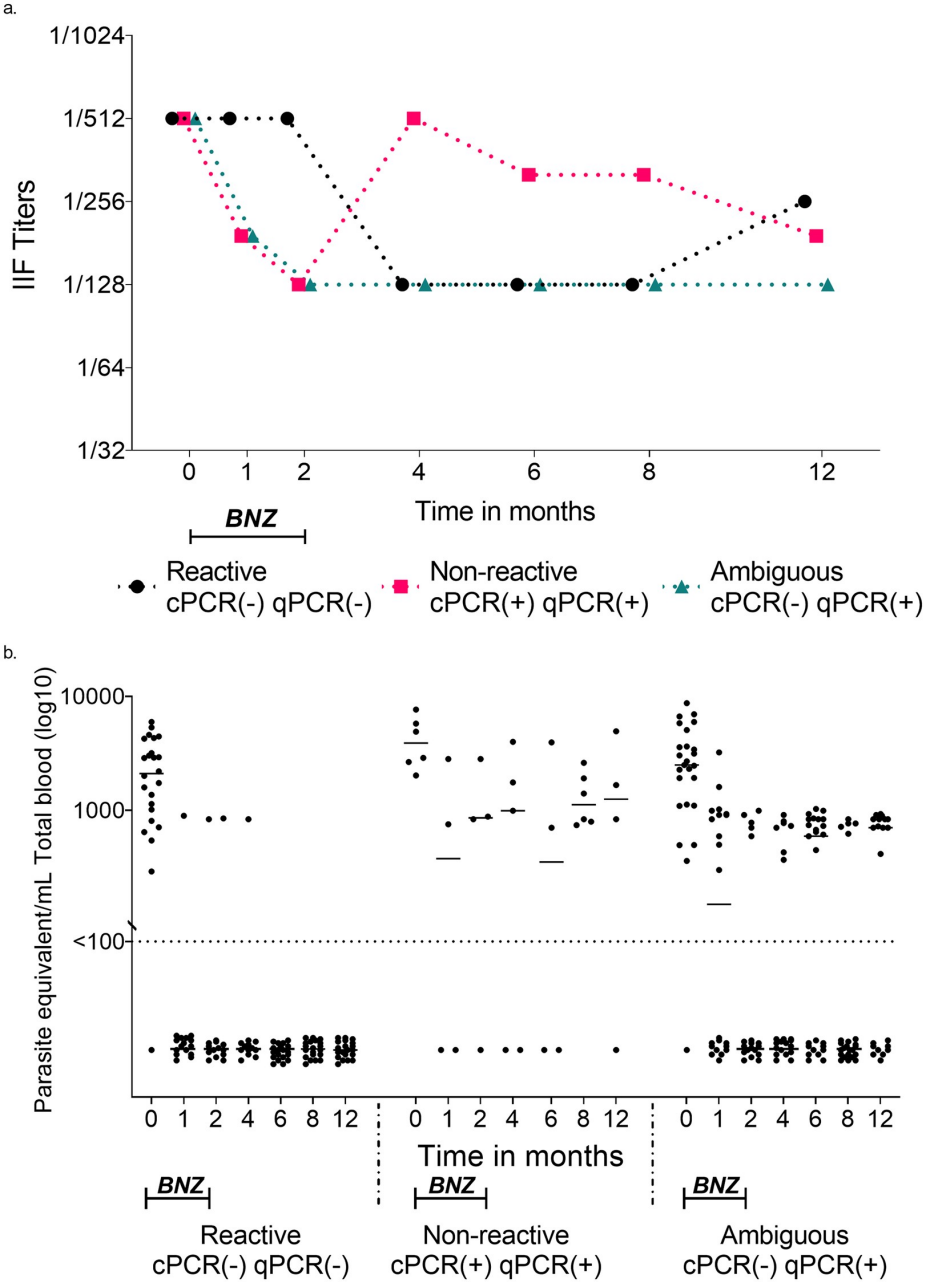
Follow-up of parasitemia and IIF titers in children according to efficacy end-points. a. IIF titers by efficacy end-points. Black circle represent the median of IIF titers of reactive group, Pink square represent the median of IIF titers of non-reactive group, and teal triangle represent the median of IIF titers of ambiguous group. b. Scatter plots: distribution of parasitic loads by efficacy end-points in each follow-up time points for 12 months. Each dot represents DNA copies of parasite equivalent in one ml of total blood by qPCR. Line represent median. Reactive group: cPCR(-) qPCR(-), (*n =* 25). Non-Reactive group: cPCR(+) qPCR(+), (*n =* 6). Ambiguous group: cPCR(-) qPCR(+), (*n =* 24). <100 = detection limit cut-off (Ct value above 45) as described in methods. qPCR, quantitative real-time PCR; IIF titers, indirect immunofluorescence Titers. BNZ, treatment with Benznidazole.

We evaluated the cPCR and qPCR results of 73 children as described in the methods section. Sixteen children were excluded because they had only one attendance during a 6–12 months period. Six children were non-reactive (cPCR(+) qPCR(+)), 26 had a total absence of *T*. *cruzi* DNA in peripheral blood by cPCR and qPCR after completion of the treatment (reactive; cPCR(-) qPCR(-)). Moreover, 25 children showed ambiguous reaction to BNZ (cPCR(-) qPCR(+)) (S4 Table). Before the treatment, the median parasitemia levels for the efficacy end-points, reactive, non-reactive and ambiguous groups were 3.3 log_10_ parasites/ml, 3.9 log_10_ parasites/ml and 3.4 log_10_ parasites/ml, respectively. No significant difference of IIF titers was observed between those groups (Fig 4a). Each efficacy end-point group showed a significant progressive reduction of parasitemia levels during follow-up (Kruskal–Wallis test; *P*<0.0001) (Fig 4b). Moreover, we compare the BNZ concentration levels between the groups and no significant difference was observed (Kruskal–Wallis test; *P* = 0.5865) (S2b Fig).

### Correlation between plasma levels of IL-17A and parasitemia

**Fig 5:**
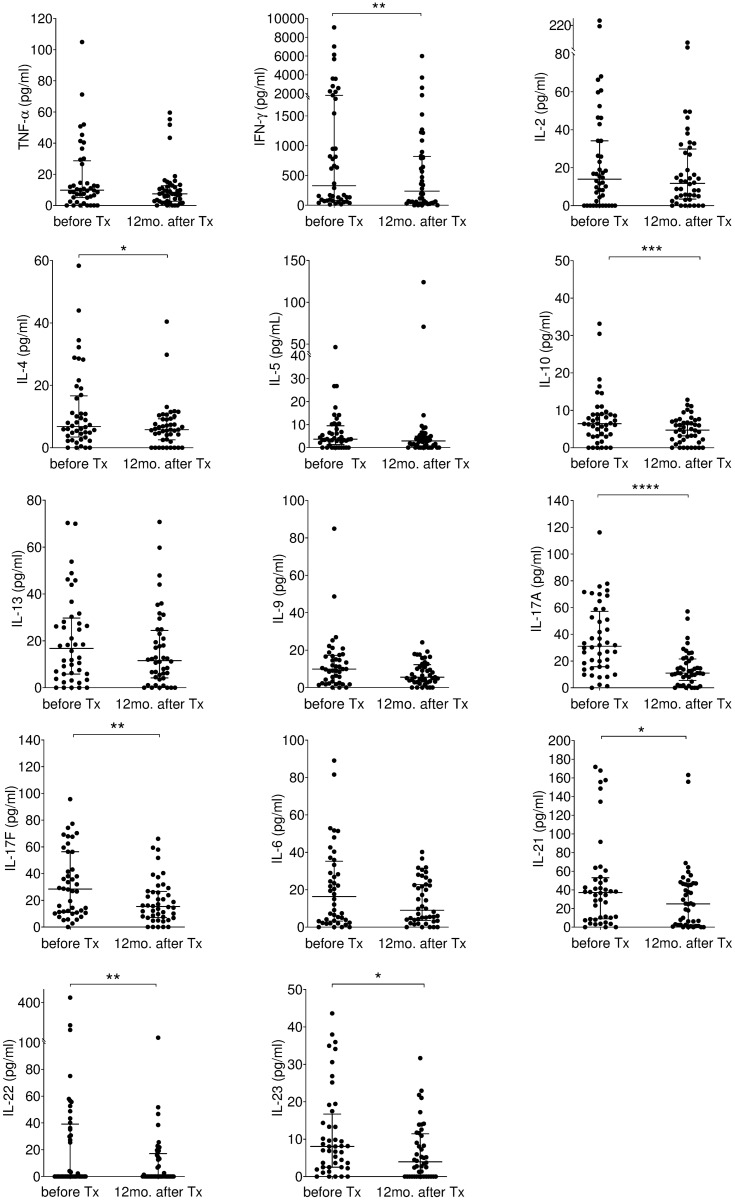
Measurement of circulating cytokine levels response. Scatter plots: distribution levels of 14 types of cytokines in children before treatment (before Tx; n = 44) and 12 months after treatment (12 mo. after Tx; n = 44). Lines represent median and IQ. Each dot represents the Interleukin mean concentration of duplicates value in a specific patient. The Wilcoxon matched-pair signed-rank test was used (*P<0.05, **P<0.01, ***P<0.001, ****P<0.0001). Tx, treatment. mo., months. IQ, Interquartile. IL, interleukin.

For monitoring cytokines before and 12 months after the treatment with BNZ, cytokines concentrations were measured as described in methods section for the plasma samples prior to BNZ treatment (n = 44) and 12 months after the treatment (n = 44). Plasma human Interleukin (IL)-10, IFN-γ, IL-4, IL-17A, IL- 17F, IL-21, IL-22 and IL-23 median concentration showed a significant decrease in children 12 months after treatment when compared with their paired samples results collected prior to treatment with BNZ (Wilcoxon matched-pairs signed-rank test; *P* = 0.0001, *P* = 0.0018, *P* = 0.0436 *P*<0.0001, *P =* 0.003, *P* = 0.0195, *P* = 0.0083, and *P* = 0.0164, respectively). No other differences were found (Fig 5). According to efficacy end-points, the reactive group showed significant decrease in IFN-γ, IL-10 and IL-17A (Wilcoxon matched-pairs signed-rank test; *P =* 0.0267, *P* = 0.0290, and *P* = 0.0010, respectively). The ambiguous group showed a similar tendency in IL-10, IL-17A and IL-17F (Wilcoxon matched-pairs signed-rank test; *P =* 0.0267, *P*<0.0001, and *P* = 0.0113, respectively). No significant differences were observed in the non-reactive group (S3 Fig).

Moreover, cytokine concentrations of the 44 plasma samples before treatment were compared to the concentrations from 16 seronegative children (healthy) confirmed by cPCR and qPCR from the same endemic area with no treatment, and we found IL-17A was significantly increased during *T*. *cruzi* infection (*P* = 0.0005), however, no changes were observed with other cytokines (S4 Fig).

**Fig 6:**
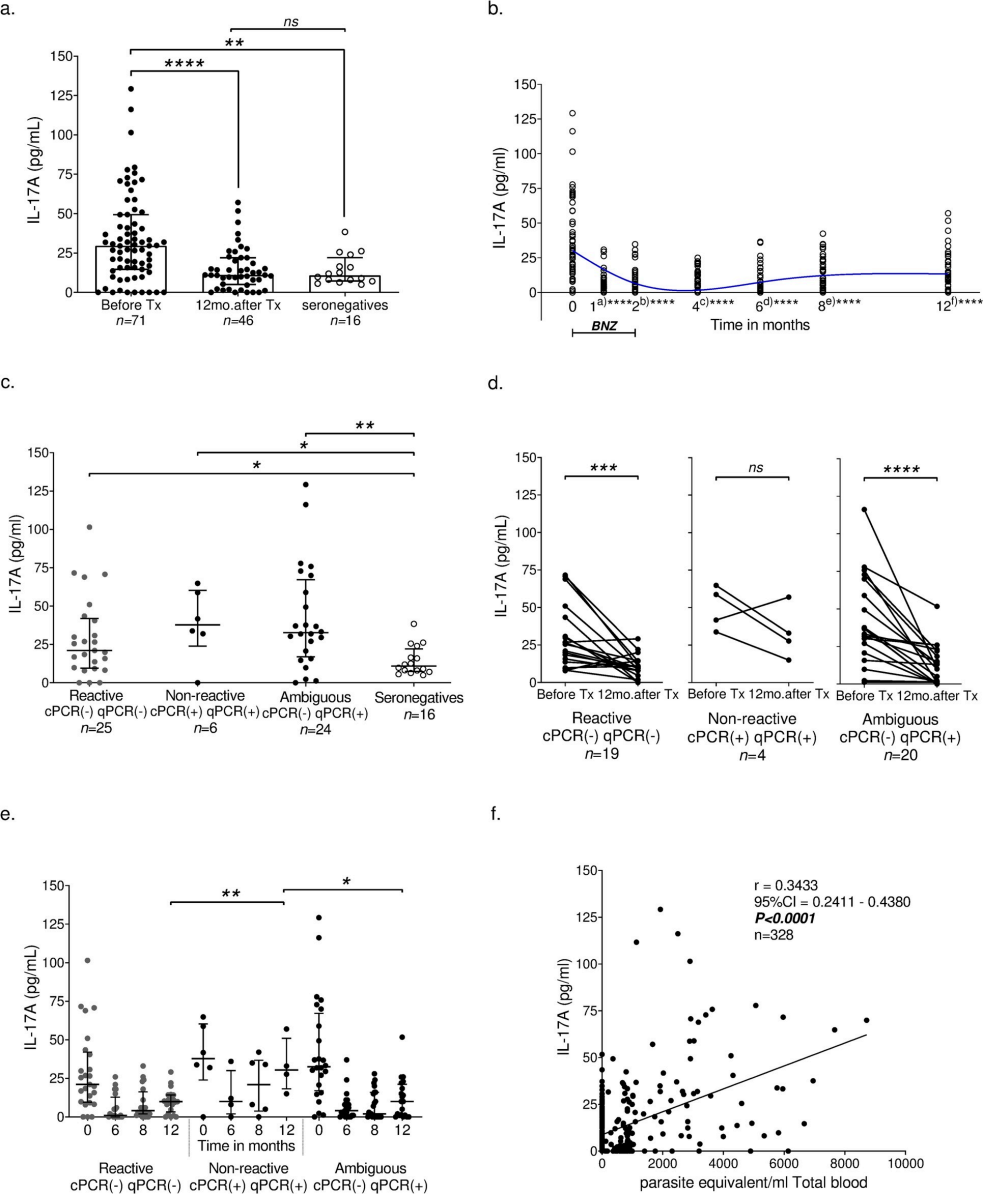
IL-17A response among recent chronic Chagas children before and post-treatment with Benznidazole. a. Scatter plots with bar: distribution levels of IL-17A in children before treatment (before Tx), 12 months after therapy (12 mo. after Tx) and seronegative individuals (seronegatives). Association was determined between two groups by the nonparametric Mann-Whitney test (**P<0.01, ****P<0.0001). b. Follow-up of distribution levels of IL-17A. Association was determined between two groups by the nonparametric Mann-Whitney test (****P<0.0001). a) before treatment versus after 30 days of treatment; b) before treatment versus after 60 days of treatment; c) before treatment versus after four months of treatment; d) before treatment versus after 6 months of treatment; e) before treatment versus after eight months of treatment; f) before treatment versus after 12 months of treatment. Loess curve is shown in color blue using a bandwidth of 1. c. Scatter plots: distribution levels of IL-17A available in children according to efficacy end-points as described in methods at before treatment (before Tx) and seronegative individuals. Association was determined between two groups by the nonparametric Mann-Whitney test (*P<0.05; **P<0.01). d. Individual patient plots for levels of IL-17A according to each efficacy end-points at before Tx. and 12mo. after Tx. *P*-values are from Wilcoxon signed rank tests (***P<0.001, ****P<0.0001). e. Scatter plots: distribution levels of IL-17A available in children according to efficacy end-points at before treatment (0) and 6, 8 and 12 months after therapy. Association was determined between two groups by the nonparametric Mann-Whitney test (*P<0.05; **P<0.01). Lines represent the median with IQ. Each dot represents the Interleukin mean concentration of duplicate values in a specific patient. f. Correlation between IL-17A levels in plasma and parasitemia by qPCR as calculated by Spearman correlation. Tx, treatment. mo., months. IQ, Interquartile. IL, interleukin. BNZ, treatment with Benznidazole.

For further evaluation of IL-17A as a marker for BNZ efficacy, we measured IL-17A for all the plasma samples available before, and during treatment and follow-up period (n = 343), and for the 16 seronegative children. As shown in Fig 6a, before the treatment, seropositive children had higher plasma levels of IL-17A (median = 29.60 pg/ml) compared to seronegative children and those high levels decreased to seronegative levels 12 months after the treatment (median = 10.82 pg/ml, *P* = 0.0023, and median = 11.02 pg/ml, *P*<0.0001, respectively) (Fig 6a). Between the initial visit and during the 12 months follow-up, the IL-17A levels decreased significantly after the treatment, similar to qPCR parasitemia measurements (Kruskal-Wallis test; *P*<0.0001) (Fig 6b, S5 Fig).

The pre- and 12 months post-therapeutic IL-17A levels were also analyzed by groups stratified with their PCR end-points (Fig 6c–6f). The levels of IL-17A were significant different between reactive, non-reactive and ambiguous groups (Kruskal-Wallis test; *P*<0.0001). We observed significant differences among the concentration levels of this cytokine during infection (before treatment) in children reactive to BNZ (cPCR(-) qPCR(-); n = 25, median = 21.05 pg/ml) compared to seronegative (n = 16, median = 10.83 pg/ml) (*P =* 0.0373), in non-reactive (cPCR(+) qPCR(+); n = 6, median = 37.75 pg/ml) compared to seronegative (n = 16, median = 10.83 pg/ml) (*P =* 0.0266), and in the ambiguous group (cPCR(-) qPCR(+); n = 24, median = 21.05 pg/ml) when compared to seronegative (*P* = 0.0373) (Fig 6c).

In addition, for children with available plasma samples from before treatment and 12 months after BNZ treatment, an individual patient analysis was performed to determine differences in concentration levels of IL-17A in non-reactive (n = 19), reactive (n = 4) and ambiguous (n = 20) groups. In Fig 6d, a significant decrease was observed after a 12-month treatment in the reactive group (*P* = 0.0010) and ambiguous group (*P*<0.0001) but not in non-reactive to BNZ group (Wilcoxon signed-rank test). In Fig 6e, before treatment and during 6–12 months follow-up, the IL-17A levels between reactive, non-reactive and ambiguous were significant different (Kruskal-Wallis test; *P*<0.0001). Moreover, after 12 months of the treatment, significant higher levels of IL-17A was observed in non-reactive group (median = 30.37pg/ml) compare to the reactive (median = 9.92 pg/ml, *P* = 0.0018) and ambiguous group (median = 10.00 pg/ml, *P* = 0.0128) (Fig 6e).

IL-17A and qPCR correlation was confirmed for all the samples tested during the study (n = 328; *r* = 0.34; *P*<0.0001) (Fig 6f). We also determined a correlation between plasma cytokine concentrations and parasitemia before treatment, finding only IL-17A and IL-17F have significant positive correlations (*r* = 0.38; *P* = 0.001 and *r* = 0.44; *P =* 0.003, respectively) (S6 Fig). At 12 months after therapy, no significant correlation was observed between plasma cytokine concentrations and parasitemia (S7 Fig).

## Discussion

Treatment cure for Chagas disease is a complex issue due to the lack of a gold standard method for verifying the achievement of a cure. Currently, since a cure means total elimination of the parasite not only from the blood but also from all tissues, there is no definitive method available [33].

In this study, although adverse events were frequently observed in around 19% of the study population, because their symptoms were very mild, mainly skin rash, the compliance was really high (one drop out from 103 treatments), showing a similar tendency with previous studies [16, 34, 35]. However, the long term follow-up after the treatment was proved difficult and we found a 25% dropout percentage of the total patients during a six months period after completion of the treatment. Finally, out of 62 informative treatment cases we found 32 BNZ reactive so-called cured (reactive group), 6 persistently infected during the follow-up period who showed high antibody levels and cPCR 121/122 minicircle kinetoplast DNA amplification as well as qPCR (non-reactive group) and 24 ambiguous reactive children defined by cPCR(-)qPCR(+) (ambiguous group).

To understand the reason why they showed different efficacy end-points, we estimated the plasma concentrations of BNZ at day 30 and 60 after initiation of the treatment (S2 Fig). Their concentration levels were within the expected effective range and we could not find any co-relation between the plasma levels of BNZ and efficacy end-points confirming that the non-cure was not due to the absence of BNZ in the plasma. However, the individuals’ data varied between 0 and 13 μg/ml (S2 Fig). There are two possible reasons to explain the variation. The first is that the duration between the time of taking medicine and of blood sampling was variable. Actually the participants took BNZ after breakfast and came to the clinic in the morning for the test without accurate record of times. The second is the possibility of high and low metabolizers in the population that might be dependent on age or genetics (36, 37). These observations open the possibility of other factors other than the pharmacokinetics and pharmacodynamics of BNZ having a significant effect on therapy, especially in the non-reactive group. Identifying the reason those six individuals were not cured will require further study of both host and parasite factors.

Although the gold standard for the diagnosis of Chagas disease is serology, it cannot be applied to the early evaluation of treatment efficacy. A 21-year follow-up cohort study with BNZ and nifurtimox as treatment for chronic Chagas patients showed that 16-year post-treatment was the average length of time for negative seroconversion [12]. Therefore, serology is difficult to use as a post-treatment monitoring tool. In this study, we found the qPCR assay was more sensitive than cPCR for the detection of circulating *T*. *cruzi* DNA. Even after cPCR results changed from positive to negative, we found around 40% were qPCR positive after a 6–12 months period. Previous studies have shown that the kinetoplast *T*. *cruzi* cPCR-based detection was useful for monitoring the infected subjects under treatment and follow-up with BNZ or nifurtimox [36, 37] yet no comparison with qPCR was evaluated. Different studies reported the qPCR as a potential for monitoring the parasitemia for treatment outcome [21, 22, 38–41]. Most recently, a study in asymptomatic *T*. *cruzi* adult carriers showed that monotherapy with BNZ was superior to posaconazole by qPCR [42]. Duffy et al. [22] followed-up for 18 months the parasitological responses to treatment with BNZ by qPCR in 38 pediatric cases with 36 having a favorable outcome, demonstrating the usefulness of qPCR assays. More recently, Bianchi et al [42] reported that 12% of treated cases persisted positive by qPCR until the end of 30 months follow-up after nifurtimox treatment in Colombian children. Our study reproduced their findings under different settings and supports the usefulness of qPCR for early detection of treatment failure although further study is necessary to observe the ambiguous group’s consequence for longer period.

In addition to the quantification of parasite DNA and specific antibody in the patient’s plasma, we investigated the plasma concentration levels of cytokines mainly produced by monocytes and lymphocytes which are supposed to be stimulated by the infected parasites [24, 43–45] to find possible biomarker(s) for the evaluation of BNZ treatment efficacy. As shown in Fig 4, IFN-γ, IL-4, IL-10, IL-17A, IL-17F, IL-21, IL-22, and IL-23 were significantly reduced after BNZ but within those the IL-17A showed the most impressive reduction. IL-17A is a pro-inflammatory cytokine secreted from activated T-cells [46]. In Chagas’ mouse model, Th17-mediated protection [47, 48] as well as pathogenicity [49, 50] have already been reported. Recently, it was discovered that through the secretion of IL-17A, Th17 cells trigger the macrophage function of NADPH oxidase as well as CD8 T-cells to provide direct protection in Chagas disease [47]. In humans, there are two major studies indicated the importance of IL-17A in the pathogenesis of cardiac Chagas [51, 52]. After the first report by Magalhães LMD et al. [53] of the association of IL-17 and better cardiac function in human Chagas disease, Sousa GR et al. [54], reported the association of IL-17A with better Left Ventricular function, suggesting the important role of IL-17A in the chronic destruction of the tissue. However, the fact that human Th17 cells are responsible for the inflammatory cell infiltration in gastrointestinal mucosa and the high expression of IL-17 in patients with inflammatory bowel disease (Ulcerative colitis and Crohn’s disease) implicates the gut residential Th17 cells’ involvement in chagasic megacolon [55–57]. Thus, Th17 and its cascade responses are one of the hot spots to study and clarify the mechanism of protection and pathogenicity of Chagas.

We found that the chronic patients showed correlation of plasma levels of IL-17A with parasitemia during infection, and they had significantly higher levels of IL-17A compared with the sero-negative controls. Those IL-17A levels decreased to normal levels after the treatment in the patients with persistent cPCR(-) qPCR(-) until 6–12 months period of follow-up. More importantly, the non-reactive group showed significantly higher levels of IL-17A 12 months after treatment, supporting the possibility of a persistent infection. However, the ambiguous group (cPCR(-) qPCR(+)) showed a significant decrease of IL-17A after 6 months indicating no persistent infection. This is compatible to the recent report that observed a decrease in the expression of IL-17 in mouse tissue after BNZ [58]. Although we could not make any clear diagnosis on whether the treated is cured or non-cured using serology and PCR, cytokine profile especially IL-17A suggested that parasite infection continued only in the non-reactive group but not in the ambiguous group (Fig 5d). Moreover, the ability of benznidazole to shift the parasite-specific T-cell response and expression of IL-17A was revealed as an early marker of *T*. *cruzi* infected children and post-therapeutic monitoring. The limitation of the present study is the small sample size, therefore its interpretation should be taken carefully. However, since qPCR is not a realistic test for monitoring the treatment efficacy, IL-17A can be applied for the evaluation treatment efficacy by ELISA, and it could facilitate the present treatment program and the development of novel drugs.

Our findings support the notion that the monitoring of appropriate immunological responses can help to understand *T*. *cruzi* infection and treatment efficacy in a short time follow-up period. To our knowledge, this is the first report describing plasma levels of IL-17A as a possible biomarker for BNZ efficacy.

## Supporting information

S1 Table Primer sets and probe sequences for qPCR. (PDF)

S2 Table Summary of positive results by ELISA, IHA, IIF, cPCR and qPCR before treatment with Benznidazole and follow-up from Santa Cruz, Bolivia. *Results are presented as median [IQR(min-max)]. ELISA and IHA Positive mean reactive when tested by Wiener Recombinant 3.0 Chagatest ELISA and Chagas Polychaco IHA, respectively. IIF titers were considered reactive when fluorescence was observed at a 1:20 or higher dilution by Immunofluor Chagas test. cPCR Positive when agarose gel show 330 bp *Trypanosoma cruzi* kDNA specific fragment amplification. qPCR Positive when *T*. *cruzi* parasite load were determined by the qPCR method as described at methods (negative by qPCR = Ct value above 45).
(PDF)

S3 Table Distribution of the patients before treatment with Benznidazole and follow-up from Santa Cruz, Bolivia. Abbreviations; Ct: Cycle threshold. IHA, Haemagglutination test. IIF, Indirect Immunofluorescence test. ELISA, Enzyme-Linked ImmunoSorbent Assay. cPCR: conventional Polymerase Chain Reaction. qPCR: Quantitative Real-Time Polymerase Chain Reaction. Absent sample in gray color. ^¶^DNA fragment analysis definitions: 1 = Reactive (cPCR(-) qPCR(-)), 2 = Non-reactive (cPCR(+) qPCR(+)), 3 = Ambiguous (cPCR(-) qPCR(+)), E = Excluded. positive = positive result tested by Wiener Recombinant 3.0 Chagatest ELISA, and Chagas Polychaco IHA test. IIF titers were considered reactive when fluorescence was observed at a 1:20 or higher dilution by Immunofluor Chagas test. cPCR Positive when agarose gel show 330 bp *Trypanosoma cruzi* kDNA specific fragment amplification. qPCR Positive when *T*. *cruzi* parasite load were determined by qPCR method as described at methods (negative by qPCR = Ct value above 45).
(XLSX)

S4 Table Efficacy end-points according to absence and presence of *T*. *cruzi* DNA fragments by cPCR and qPCR. Abbreviations; cPCR: conventional Polymerase Chain Reaction. qPCR: Quantitative Real-Time Polymerase Chain Reaction. ^¶^months after treatment. + = positive,— = negative.
(PDF)

S5 Table STROBE statement—Checklist for the study title: IL-17A, a possible biomarker for the evaluation of treatment response in *Trypanosoma cruzi* infected children: A 12-months follow-up study in Bolivia. (PDF)

S1 Fig Chromatograms of plasma spiked with the retention times of Benznidazole (3.635 minutes) and Diazepam (6.003 minutes). (TIF)

S2 Fig Benznidazole concentration levels among recent chronic Chagas children. a. Plasma samples available from each seropositive patient before treatment (0, n = 65), 30 days (n = 56) and 60 days (n = 41) days after the initiation of treatment (3-time points) were analyzed using high-performance liquid chromatography. b. BNZ concentration by efficacy end-points at 30 days after the initiation of treatment. Lines represent median and IQ. Each dot represents BNZ concentration levels (μg/ml). BNZ; benznidazole.
(TIF)

S3 Fig Measurement of circulating cytokine levels by efficacy end-points. Scatter plots: distribution levels of 14 types of cytokines by efficacy end-points. Reactive group: cPCR(-)qPCR(-), Non-Reactive group: cPCR(+) qPCR(+), Ambiguous group: cPCR(-)qPCR(+), as described in methods. Lines represent median and IQ. Each dot represents the Interleukin mean concentration of duplicates value in a specific patient. Association was determined between two groups by the nonparametric Mann-Whitney test (*P<0.05, ***P<0.001, ****P<0.0001). Tx, treatment with benznidazole. IQ, Interquartile. IL, interleukin.
(TIF)

S4 Fig Measurement of circulating cytokine levels as compared to seronegative individuals. Scatter plots: distribution levels of 14 types of cytokines in children before treatment (before Tx; n = 44) and seronegative children from the same endemic area (Seronegative individuals; n = 16). Lines represent median and IQ. Each dot represents the Interleukin mean concentration of duplicates value in a specific patient. Association was determined between two groups by the nonparametric Mann-Whitney test (***P<0.001). Tx, treatment with benznidazole. IQ, Interquartile. IL, interleukin.
(TIF)

S5 Fig IL-17A levels before and during follow-up among recent chronic Chagas children. Scatter plots with bar: distribution levels of IL-17A in children in each follow-up time points for 12 months. Association was determined between two groups by the nonparametric Mann-Whitney test (****P<0.0001). Lines represent median and IQ. Each dot represents the plasma concentration of IL-17A in each child. IQ, Interquartile. IL, interleukin.
(TIF)

S6 Fig Correlation between cytokine levels in plasma and parasitemia by qPCR before treatment as calculated by Spearman correlation. IL, interleukin. r, Spearman r. *P*, P value.
(TIF)

S7 Fig Correlation between cytokine levels in plasma and parasitemia by qPCR after 12 months of treatment as calculated by Spearman correlation. IL, interleukin. r, Spearman r. *P*, P value.
(TIF)
