# Supplementary material for: IL-17A, a possible biomarker for the evaluation of treatment response in Trypanosoma cruzi infected children: A 12-months follow-up study in Bolivia
Source: PLoS Negl Trop Dis. 2019 Sep 25;13(9):e0007715. doi: 10.1371/journal.pntd.0007715 (PMC6760767; doi:10.1371/journal.pntd.0007715)
Supplement: S1 Table — (PDF) [file pntd.0007715.s001.pdf]

S1 Table. Primer sets and probes sequences for qPCR

PCR	Primer or Probe	Sequence (5'-3')	Concentration
Real-time *T. cruzi* satellite K01773.1	TCZNTAQF (forward)	GCTCTTGCCCACACGGGTG	26.3 µM
	TCZNTAQR (reverse)	AAGCAGCGGATAGTTCAGGGT	21.0 µM
	TCZ-FAM (probe)	ACTCGGCTGATCGTT	24.3 µM
Real-time Internal control GAPDH-Intron C	GAPDH-C-36F (forward)	GCCCCTTCATACCCTCACGTA	29.5 µM
	GAPDH-C-141R (reverse)	TGACAAGCTTCCCGTTCTCAG	27.8 µM
			32.4 µM
	GAPDH-VIC (probe)	ATGTTCCAATATGATTCCAC	11.2 µM
