# Supplementary material for: IL-17A, a possible biomarker for the evaluation of treatment response in Trypanosoma cruzi infected children: A 12-months follow-up study in Bolivia
Source: PLoS Negl Trop Dis. 2019 Sep 25;13(9):e0007715. doi: 10.1371/journal.pntd.0007715 (PMC6760767; doi:10.1371/journal.pntd.0007715)
Supplement: S2 Table — *Results are presented as median [IQR(min-max)]. ELISA and IHA Positive mean reactive when tested by Wiener Recombinant 3.0 Chagatest ELISA and Chagas Polychaco IHA, respectively. IIF titers were considered reactive when fluorescence was observed at a 1:20 or higher dilution by Immunofluor Chagas test. cPCR Positive when agarose gel show 330 bp Trypanosoma cruzi kDNA specific fragment amplification. qPCR Positive when T. cruzi parasite load were determined by the qPCR method as described at methods (negative by qPCR = Ct value above 45). (PDF) [file pntd.0007715.s002.pdf]

S2 Table. Summary of positive results by ELISA, IHA, IIF, cPCR and qPCR before treatment with Benznidazole and follow-up from Santa Cruz, Bolivia.

Group	Total *n*	ELISA	IHA	IIF		cPCR	qPCR	
				Positive *n* (%)	Titers		Positive *n* (%)	parasites/ml Total blood (log_{10})
		Positive *n* (%)	Positive *n* (%)		Median [IQR(min - max)]*	Positive *n* (%)		Median [IQR(min - max)]*
Before Treatment	71	71 (100)	71 (100)	71 (100)	1/512 [1/256 - 1/512]	63 (89)	67 (94)	3.39 [3.05 - 3.63]
After 30 days of treatment	52	51 (98)	51 (98)	51 (98)	1/512 [1/128 - 1/512]	8 (85)	16 (31)	2.96 [2.79 - 3.01]
After 60 days of treatment	43	42 (98)	42 (98)	42 (98)	1/512 [1/128 - 1/512]	3 (7)	11 (25)	2.92 [2.88 - 2.96]
After 4 months of treatment	48	47 (90)	47 (90)	47 (90)	1/128 [1/128 - 1/512]	4 (8)	12 (25)	2.90 [2.68- 2.99]
After 6 months of treatment	54	53 (98)	53 (98)	53 (98)	1/128 [1/128 - 1/384]	1 (2)	16 (30)	2.90 [2.81 - 2.96]
After 8 months of treatment	54	53 (98)	53 (98)	53 (98)	1/128 [1/128 - 1/512]	5 (9)	11 (20)	2.90 [2.87 - 3.15]
After 12 months of treatment	46	45 (98)	45 (98)	45 (98)	1/256 [1/128 - 1/512]	2 (4)	15 (33)	2.92 [2.85 - 2.96]

*Results are presented as median [IQR(min-max)]

ELISA and IHA Positive mean reactive when tested by Wiener Recombinant 3.0 Chagatest ELISA and Chagas Polychaco IHA, respectively.

IIF titers were considered reactive when fluorescence was observed at a 1:20 or higher dilution by Immunofluor Chagas test.

cPCR Positive when agarose gel show 330 bp *Trypanosoma cruzi* kDNA specific fragment amplification.

qPCR Positive when *T. cruzi* parasite load were determined by the qPCR method as described at methods (Ct value below 45).
