# Supplementary material for: IL-17A, a possible biomarker for the evaluation of treatment response in Trypanosoma cruzi infected children: A 12-months follow-up study in Bolivia
Source: PLoS Negl Trop Dis. 2019 Sep 25;13(9):e0007715. doi: 10.1371/journal.pntd.0007715 (PMC6760767; doi:10.1371/journal.pntd.0007715)
Supplement: S4 Table — Abbreviations; cPCR: conventional Polymerase Chain Reaction. qPCR: Quantitative Real-Time Polymerase Chain Reaction. ¶months after treatment. + = positive,— = negative. (PDF) [file pntd.0007715.s004.pdf]

S4 Table. Efficacy end-points according to absence and presence of *T.cruzi* DNA fragments by cPCR and qPCR

	cPCR			**qPCR**			Total
	6¶	8¶	12¶	6¶	8¶	12¶	*n*=73
Reactive cPCR(-)qPCR(-) *n*=26	-	-	-	-	-	-	8
	-	-		-	-		5
	-		-	-		-	6
		-	-		-	-	7
Non-reactive cPCR(+) qPCR (+) *n*=6	+	+	+	+	+	+	1
		+			+		2
	-	+	-	-	+	+	1
	-	-	+	+	+	+	1
	-	+	-	-	+	-	1
Ambiguos cPCR(-)qPCR(+) *n*=25	-	-	-	+	+	+	1
	-	-	-	+	+	-	2
	-	-	-	+	-	-	6
	-	-	-	+	-	+	2
	-	-	-	-	-	+	8
	-	-	-	-	+	-	2
	-	-		+	+		1
	-	-		+	-		2
	-		-	-		+	1
Excluded *n*=16	-			-			6
		-			-		5
			-			-	1
							4

cPCR: conventional Polymerase Chain Reaction

qPCR: Quantitative Real-Time Polymerase Chain Reaction

¶months after treatment, + = positive, - = negative
