# Supplementary material for: IL-17A, a possible biomarker for the evaluation of treatment response in Trypanosoma cruzi infected children: A 12-months follow-up study in Bolivia
Source: PLoS Negl Trop Dis. 2019 Sep 25;13(9):e0007715. doi: 10.1371/journal.pntd.0007715 (PMC6760767; doi:10.1371/journal.pntd.0007715)
Supplement: S5 Table — (PDF) [file pntd.0007715.s005.pdf]

Table S5. STROBE Statement—Checklist for the study title: IL-17A, a possible biomarker for the evaluation of treatment response in *Trypanosoma cruzi* infected children: A 12-months follow-up study in Bolivia

	Item No	Recommendation	Page no.	Relevant text from Manuscript
**Title and abstract**	1	(*a*) Indicate the study's design with a commonly used term in the title or the abstract	1	Full title
		(*b*) Provide in the abstract an informative and balanced summary of what was done and what was found	4	Abstract, *Methods and Findings* section
**Introduction**				
Background/rationale	2	Explain the scientific background and rationale for the investigation being reported	7-8	Paragraphs 1-5
Objectives	3	State specific objectives, including any prespecified hypotheses	8	Paragraph 6
**Methods**				
Study design	4	Present key elements of study design early in the paper	9	Methods, *Recruitment and participants* section.
Setting	5	Describe the setting, locations, and relevant dates, including periods of recruitment, exposure, follow-up, and data collection	9-10	Methods, *Recruitment and participants* section. *Samples and DNA extraction* section, paragraph 1
Participants	6	Give the eligibility criteria, and the sources and methods of selection of participants. Describe methods of follow-up	9-10<br>Fig 1	Methods, *Recruitment and participants* section*:* paragraph 2: *the inclusion criteria…,* paragraph 3 and 4. Fig 1. *The flow diagram of study design, patient selection, treatment and follow-up*
Variables	7	Clearly define all outcomes, exposures, predictors, potential confounders, and effect modifiers. Give diagnostic criteria, if applicable	9, 14	Methods, *Recruitment and participants* section. *Efficacy End-points* section.
Data sources/ measurement	8	For each variable of interest, give sources of data and details of methods of assessment (measurement). Describe comparability of assessment methods if there is more than one group	14-15	Methods, *Efficacy End-points* section. *Statistical analysis* section
Bias	9	Describe any efforts to address potential sources of bias	10-14	Methods, *Recruitment and participants* section*:* paragraph 4. *Quantitative Real Time PCR section, Efficacy End-points* section.
Study size	10	Explain how the study size was arrived at	9<br>Fig 1	Methods, *Recruitment and participants* section. Fig 1. *The flow diagram of study design, patient selection, treatment and follow-up*
Quantitative variables	11	Explain how quantitative variables were handled in the analyses. If applicable, describe which groupings were chosen and why	14-15	Methods, *Efficacy End-points* section. *Statistical analysis* section
Statistical methods	12	(*a*) Describe all statistical methods, including those used to control for confounding	15	Methods, *Statistical analysis* section

		(*b*) Describe any methods used to examine subgroups and interactions	14-15	Methods, *Efficacy End-points* section. *Statistical analysis* section
		(*c*) Explain how missing data were addressed	14	Methods, *Efficacy End-points* section.
		(*d*) If applicable, explain how loss to follow-up was addressed	14	Methods, *Efficacy End-points* section.
		(*e*) Describe any sensitivity analyses	12-15	Methods, *Quantitative Real Time PCR* section. *Efficacy End-points* section. *Statistical analysis* section
**Results**				
Participants	13	(a) Report numbers of individuals at each stage of study—eg numbers potentially eligible, examined for eligibility, confirmed eligible, included in the study, completing follow-up, and analysed	16<br>36, Fig 1	Results, *Patients' characteristics* section. Fig 1. *The flow diagram of study design, patient selection, treatment and follow-up*
		(b) Give reasons for non-participation at each stage	16<br>36, Fig 1	Results, *Patients' characteristics* section, last paragraph. Fig 1. *The flow diagram of study design, patient selection, treatment and follow-up*
		(c) Consider use of a flow diagram	36, Fig 1	Fig 1. *The flow diagram of study design, patient selection, treatment and follow-up*
Descriptive data	14	(a) Give characteristics of study participants (eg demographic, clinical, social) and information on exposures and potential confounders	16	Results, *Patients' characteristics* section, first paragraph.
		(b) Indicate number of participants with missing data for each variable of interest	16<br>36, Fig 1	Results, *Patients' characteristics* section, last paragraph. Fig 1. *The flow diagram of study design, patient selection, treatment and follow-up*
		(c) Summarise follow-up time (eg, average and total amount)	16	Results, *Patients' characteristics* section, last paragraph. Fig 1. *The flow diagram of study design, patient selection, treatment and follow-up*
Outcome data	15	Report numbers of outcome events or summary measures over time	16-21.<br>S3 Table	Results, *Side effects and plasma level of BNZ* section, *One-year follow-up of T.cruzi DNA fragments* section, *Correlation between plasma levels of IL-17A and parasitemia* section. Supplementary tables: *S3 Table. Distribution of the patients before treatment with Benznidazole and follow-up from Santa Cruz, Bolivia*
Main results	16	(*a*) Give unadjusted estimates and, if applicable, confounder-adjusted estimates and their precision (eg, 95% confidence interval). Make clear which confounders were adjusted for and why they were included	16-21.<br>36-39.<br>Figs 2-6<br>S2-S4 Tables	Results, *Side effects and plasma level of BNZ* section, *One-year follow-up of T.cruzi DNA fragments* section, *Correlation between plasma levels of IL-17A and parasitemia* section. Figs 3-5. S2-S4 Tables
Other analyses	17	Report other analyses done—eg analyses of subgroups and interactions, and sensitivity analyses	S2-S7 figs	Supplementary figures from S2-S7 figs
**Discussion**				
Key results	18	Summarise key results with reference to study objectives	22-25	Discussion section

Limitations	19	Discuss limitations of the study, taking into account sources of potential bias or imprecision. Discuss both direction and magnitude of any potential bias	22, 25	Discussion section, paragraph 2, 6
Interpretation	20	Give a cautious overall interpretation of results considering objectives, limitations, multiplicity of analyses, results from similar studies, and other relevant evidence	22-25	Discussion section
Generalisability	21	Discuss the generalisability (external validity) of the study results	23-25	Discussion section
**Other information**				
Funding	22	Give the source of funding and the role of the funders for the present study and, if applicable, for the original study on which the present article is based		Funding section
