# Supplementary figures and images for: IL-17A, a possible biomarker for the evaluation of treatment response in Trypanosoma cruzi infected children: A 12-months follow-up study in Bolivia
Source: PLoS Negl Trop Dis. 2019 Sep 25;13(9):e0007715. doi: 10.1371/journal.pntd.0007715 (PMC6760767; doi:10.1371/journal.pntd.0007715)

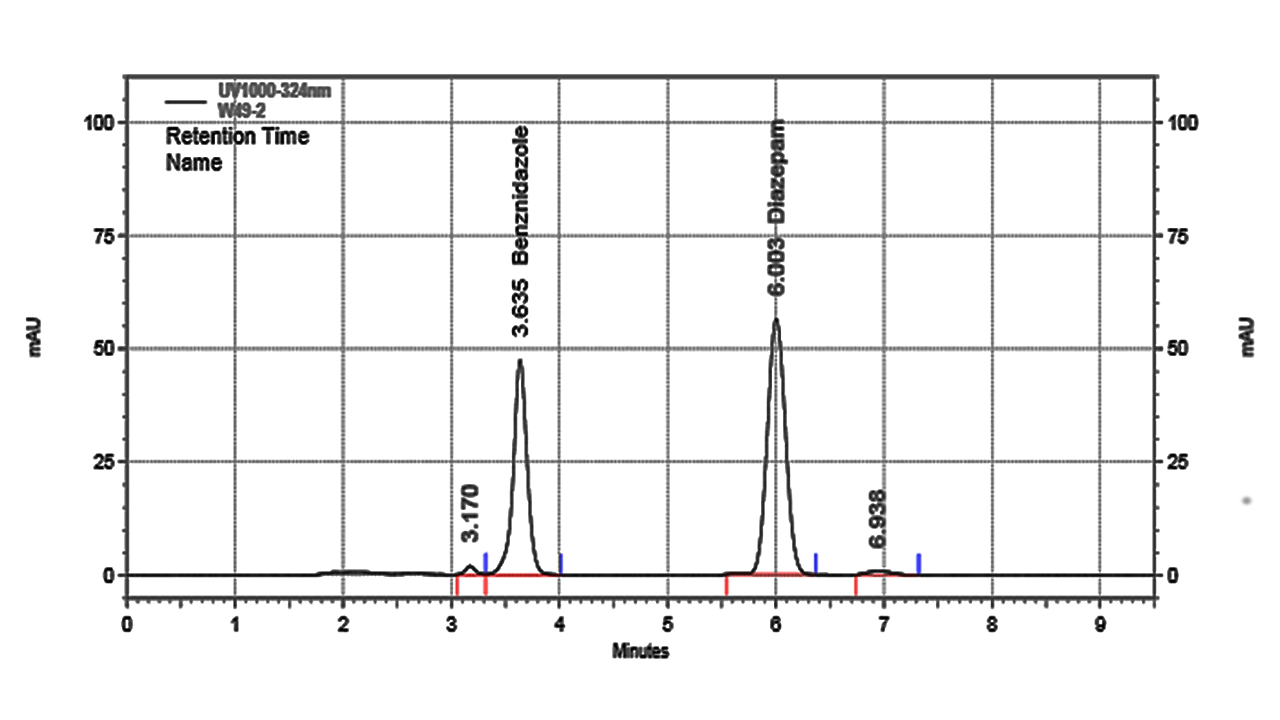

Supplement: S1 Fig — (TIF) [file pntd.0007715.s006.tif]

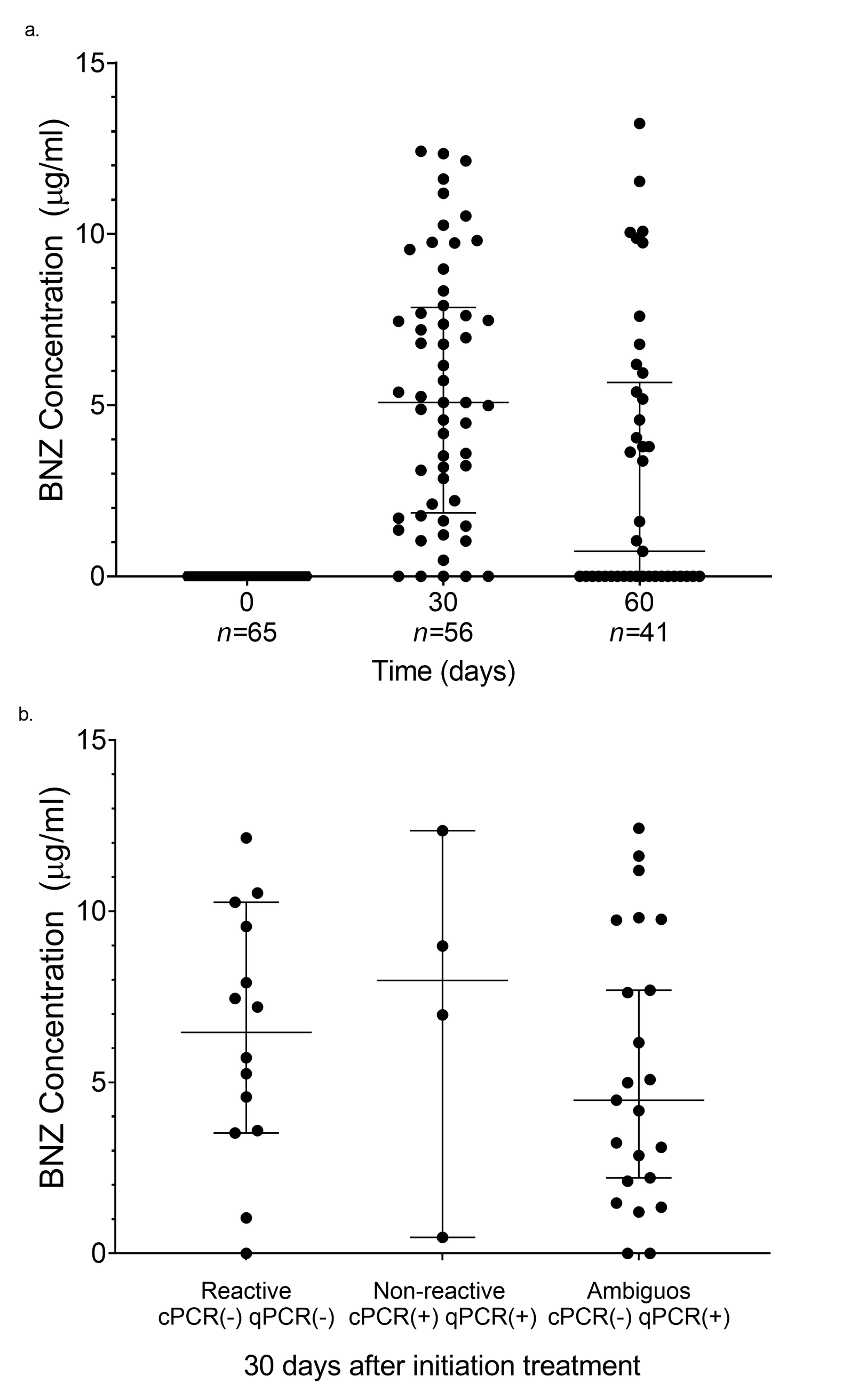

Supplement: S2 Fig — a. Plasma samples available from each seropositive patient before treatment (0, n = 65), 30 days (n = 56) and 60 days (n = 41) days after the initiation of treatment (3-time points) were analyzed using high-performance liquid chromatography. b. BNZ concentration by efficacy end-points at 30 days after the initiation of treatment. Lines represent median and IQ. Each dot represents BNZ concentration levels (μg/ml). BNZ; benznidazole. (TIF) [file pntd.0007715.s007.tif]

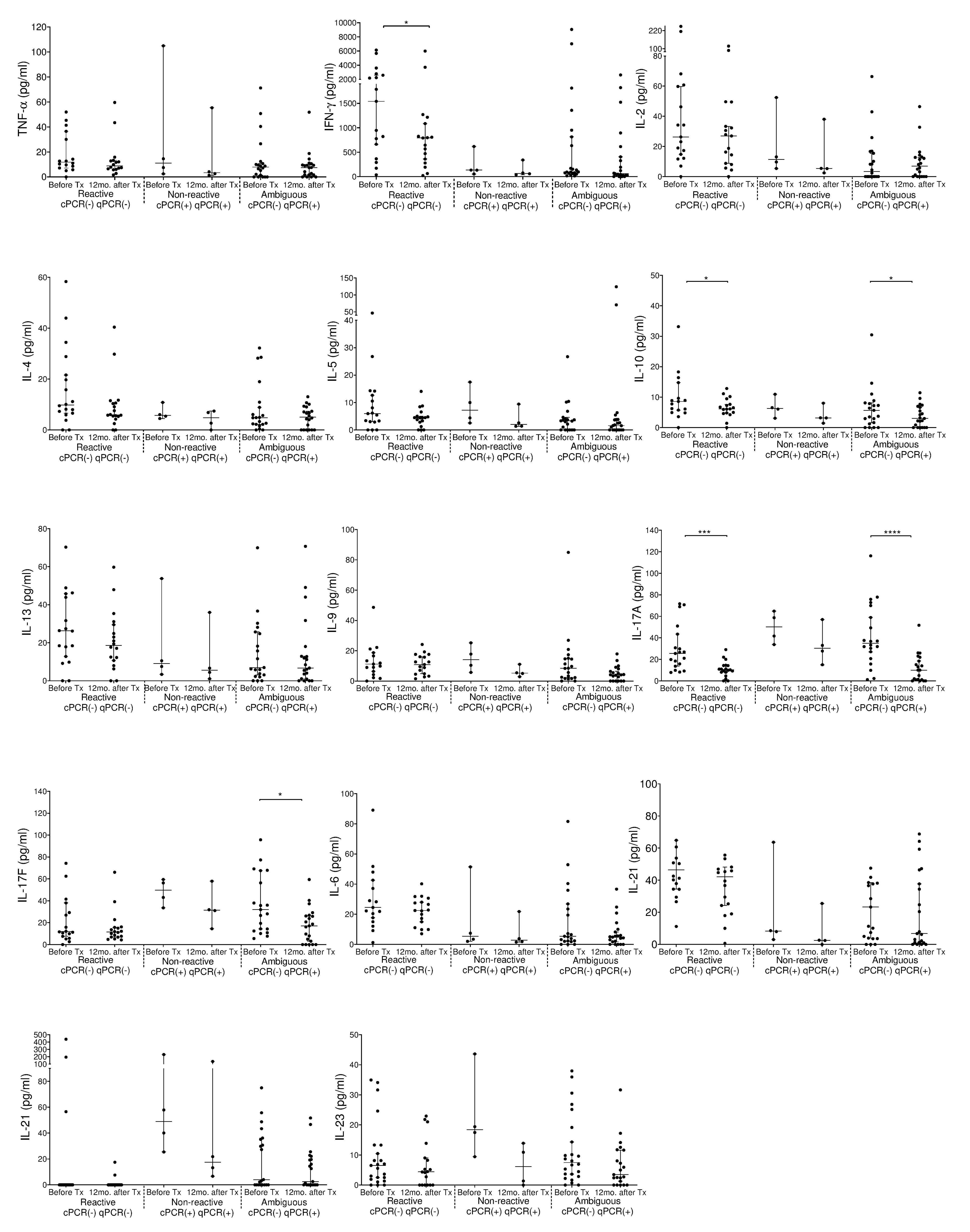

Supplement: S3 Fig — Scatter plots: distribution levels of 14 types of cytokines by efficacy end-points. Reactive group: cPCR(-)qPCR(-), Non-Reactive group: cPCR(+) qPCR(+), Ambiguous group: cPCR(-)qPCR(+), as described in methods. Lines represent median and IQ. Each dot represents the Interleukin mean concentration of duplicates value in a specific patient. Association was determined between two groups by the nonparametric Mann-Whitney test (*P<0.05, ***P<0.001, ****P<0.0001). Tx, treatment with benznidazole. IQ, Interquartile. IL, interleukin. (TIF) [file pntd.0007715.s008.tif]

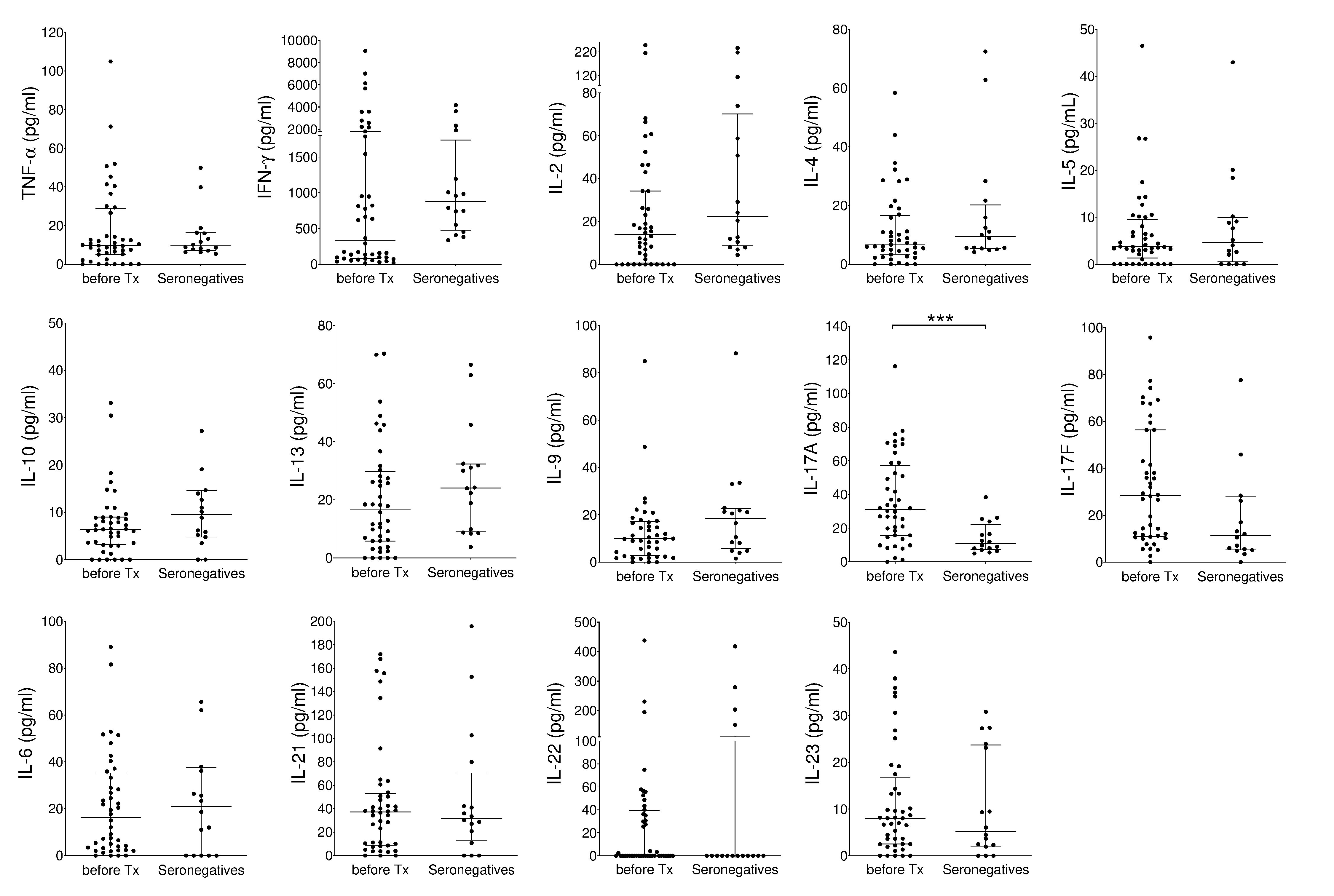

Supplement: S4 Fig — Scatter plots: distribution levels of 14 types of cytokines in children before treatment (before Tx; n = 44) and seronegative children from the same endemic area (Seronegative individuals; n = 16). Lines represent median and IQ. Each dot represents the Interleukin mean concentration of duplicates value in a specific patient. Association was determined between two groups by the nonparametric Mann-Whitney test (***P<0.001). Tx, treatment with benznidazole. IQ, Interquartile. IL, interleukin. (TIF) [file pntd.0007715.s009.tif]

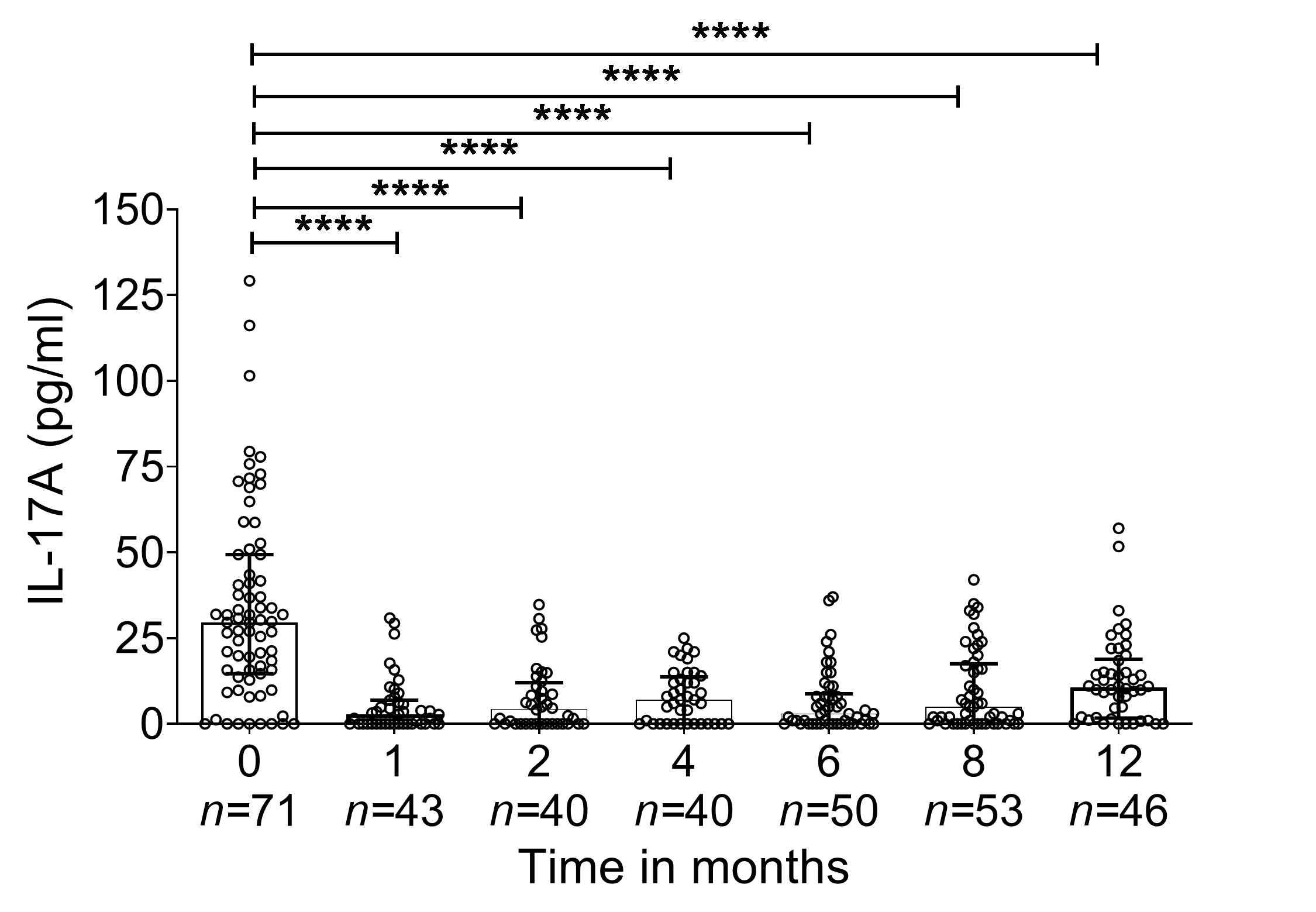

Supplement: S5 Fig — Scatter plots with bar: distribution levels of IL-17A in children in each follow-up time points for 12 months. Association was determined between two groups by the nonparametric Mann-Whitney test (****P<0.0001). Lines represent median and IQ. Each dot represents the plasma concentration of IL-17A in each child. IQ, Interquartile. IL, interleukin. (TIF) [file pntd.0007715.s010.tif]

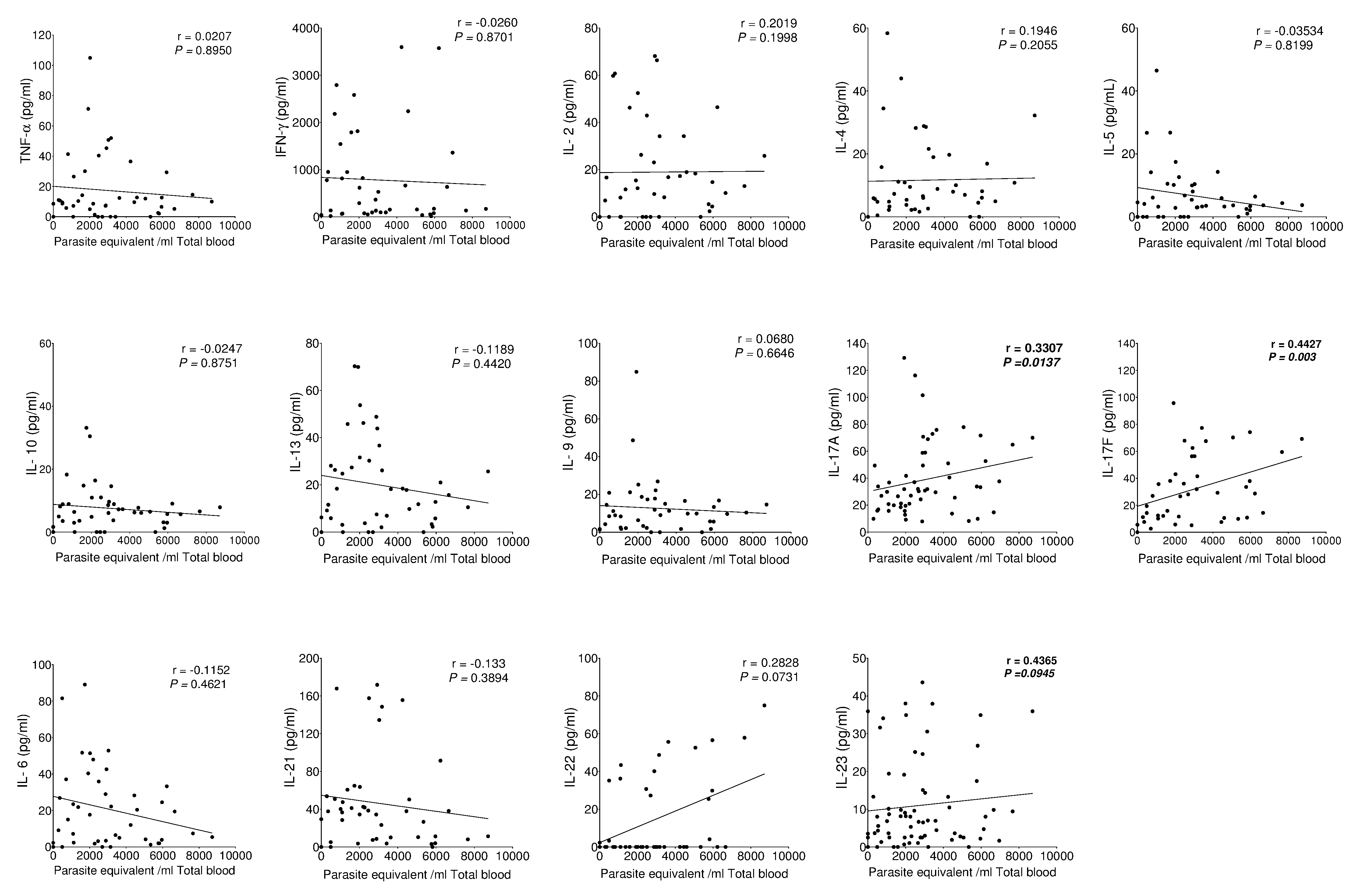

Supplement: S6 Fig — IL, interleukin. r, Spearman r. P, P value. (TIF) [file pntd.0007715.s011.tif]

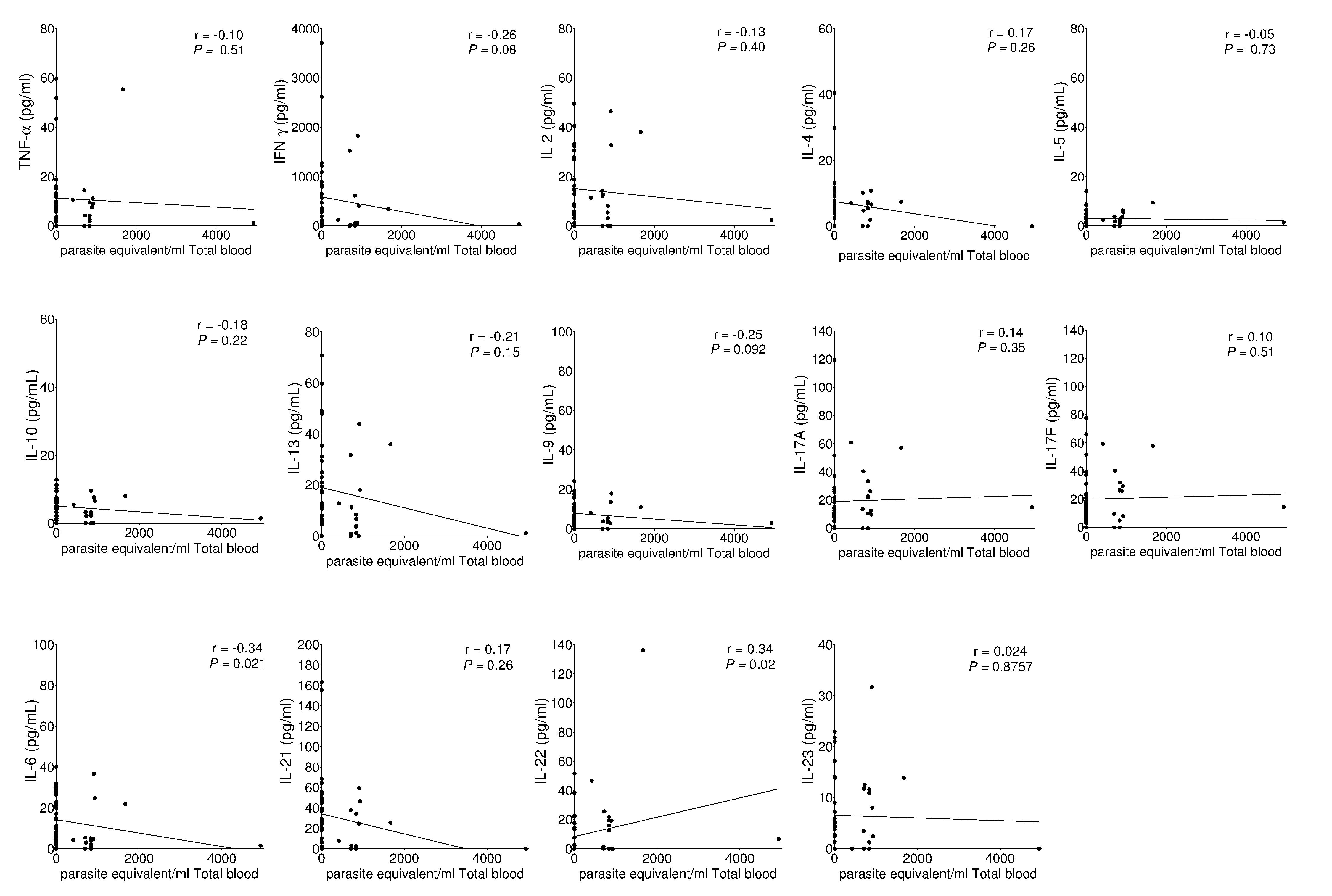

Supplement: S7 Fig — IL, interleukin. r, Spearman r. P, P value. (TIF) [file pntd.0007715.s012.tif]
